# Calcium trafficking in the mussel *Mytilus galloprovincialis* suggests conserved biomineralization pathways

**DOI:** 10.1038/s42003-026-10210-2

**Published:** 2026-05-11

**Authors:** Ernesto Ruiz-Villaespesa, Antonio G. Checa, Xiaoyan Li, Carmen Salas, Marta de Frutos

**Affiliations:** 1 Departamento de Estratigrafía y Paleontología, Facultad de Ciencias, Universidad de Granada, Granada, Spain; 2 Laboratoire de Physique des Solides (LPS), CNRS UMR 8502, Université Paris-Saclay, Orsay, France; 3 Departamento de Biología Animal, Facultad de Ciencias, Universidad de Málaga, Málaga, Spain

**Keywords:** Cell biology, Chemical biology

## Abstract

Vesicles play a key role in biomineralization pathways by mediating ion concentration and transport while maintaining cellular homeostasis. Whereas calcium-loaded vesicles have been extensively studied in organisms such as coccolithophores and vertebrates, comparatively little is known about those involved in mollusc shell mineralization. Using electron microscopy and electron energy loss spectroscopy (EELS), we analyzed the ultrastructure and composition of three vesicle types predominant near the shell’s growth surface in cryofixed juvenile *Mytilus galloprovincialis*: bilayered vesicles, ribosome-associated vesicles (RAVs), and mitochondria containing electron-dense granules. Calcium associated with organic compounds was detected in all of them. Mitochondrial granules were shown to be composed of an amorphous mixed phase containing substantial amounts of calcium and phosphorus, in addition to organic compounds. Identical Ca- and P-rich granules have been widely reported in the mitochondria of bone-related cells, where their presence is associated with the regulation of intracellular calcium and the initiation of hydroxyapatite formation. Their presence in calcifying cells of both molluscs and vertebrates, together with increasing evidence of phosphate involvement in the regulation of transient calcium carbonate precursors, leads us to propose a conserved pathway for calcium transport, concentration and storage across phyla, regardless of the final mineral phase.

## Abstract (web summary)

STEM–EELS analysis of calcium-loaded vesicles in cryofixed *Mytilus galloprovincialis* suggests a potentially conserved pathway for calcium transport, concentration, and storage across biomineralizing phyla, linked to intracellular calcium regulation mechanisms.

## Introduction

Biomineralization pathways encompass the uptake, transport, storage and deposition of the ions used by living organisms to produce mineral structures^1,2^. Calcium routes are particularly significant due to the prevalence and abundance of calcium-based biominerals. The most abundant are calcium carbonate minerals, which predominate in a wide variety of taxa, mainly as calcite and/or aragonite, forming structures with different functionalities (e.g.: protection, skeleton, gravity sensing). On the other hand, calcium phosphate minerals, though to a lesser extent than calcium carbonates, are also present in many groups, including the carbonate hydroxyapatites of vertebrate skeletons^3,4^.

The regulation of calcium, phosphate and carbonate ion concentrations is essential for the correct functioning of the cells. Many cellular activities require calcium and phosphate as messengers^5,6^ and their imbalance can lead to cell apoptosis or necrosis^7^. To maintain cytosolic homeostasis, membrane-bound organelles are used as compartments to concentrate, store and transport the high amounts of ions required for mineralization^2,8^.

Vesicle-mediated calcium concentration, transport and deposition have been reported in a great variety of biomineralizing groups^2,9^, including: foraminifera^10^, coccolithophores^11^, corals^12^, sea urchins^8^, molluscs^13,14^ and vertebrates^15,16^. In certain cases, such as in coccolithophores, intracellular crystallization occurs within specialized vesicles^11,17^. In others, ions or amorphous nanoparticles are transported by the vesicles to a biologically controlled extracellular compartment, where crystallization takes place^2,9^.

The role of amorphous precursor phases in the formation of calcareous structures has been under investigation for quite some time^18–20^. Over a century later, amorphous calcium carbonate (ACC) or amorphous calcium phosphate (ACP) have been identified in a wide variety of biologically calcified structures across species from different phyla, where they function either as stable phases or as transient precursors to crystalline phases^21–29^. Most evidence of amorphous transient precursors comes from the grainy texture, interpreted as remains of amorphous nanoparticles deposited over the growing crystal surfaces. This surface nanoroughness reflects growth through non-classical crystallization by particle attachment (crystallization by particle attachment, CPA, hypothesis)^30–33^. Growth by ion attachment is not excluded and would occur simultaneously in order to fill the interstices left by the attached nanoparticles^9,31,34^. Alternatively, the lumps that generate surface nanoroughness may not be the original amorphous particles, but irregularities of the mineralization front created during the crystallization process^35,36^. Indeed, as seen in the giant barnacle *Austromegabalanus psittacus*, these lumps appear to represent the recently crystallized zones, surrounded by a discontinuous amorphous cortex made of an organic-rich phase containing ACC^36^.

Despite these findings, direct evidence tracing the journey of mineral precursors from the mollusc mantle cells to the shell’s growing surface is scarce^14,37^. Even if vesicle-mediated transport appears to be a common mechanism^9^, the characterization of these vesicles, their cargo and their relationship with other organelles remains to be clarified. In this article, we describe the structure and chemical composition of vesicles located near the shell’s growth surface in vitrified juvenile *Mytilus galloprovincialis* (Lamarck, 1819), using a combination of electron microscopy and spectroscopy techniques. Furthermore, we report the presence of Ca- and P-rich mitochondrial granules in a mineralizing tissue involved in the formation of a calcium carbonate shell. These CaP granules have been largely described in bone-related cells^16,38–47^, where they seem to play a role in the formation of bone hydroxyapatite^44–47^. This finding, together with the increasing evidence for the involvement of phosphate in regulating calcium carbonate-based systems^2,48^, leads us to propose the existence of conserved biomineralization pathways from invertebrates to vertebrates, rooted in an ancestral mechanism of intracellular calcium regulation.

## Results and discussion

**Fig. 1:**
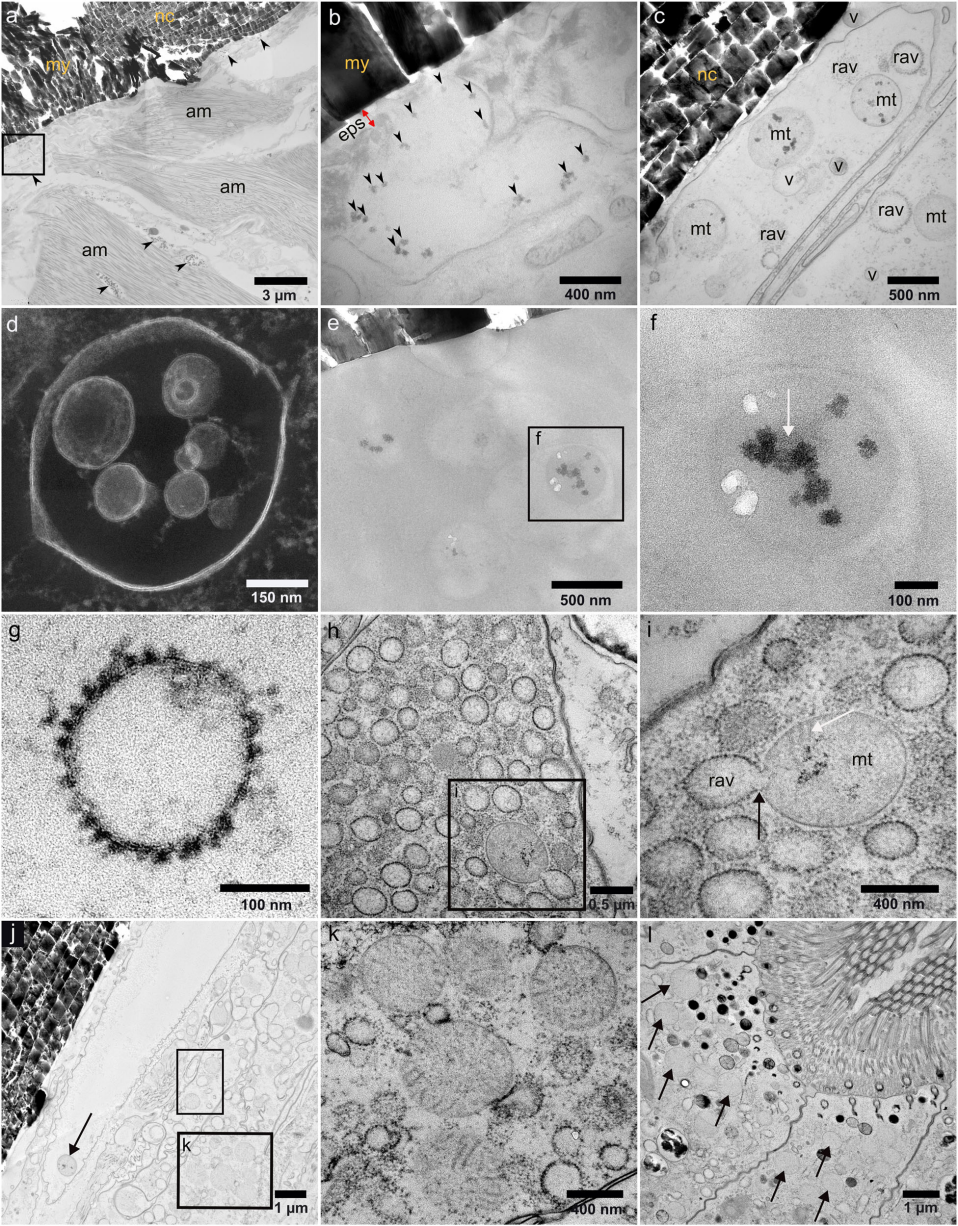
TEM images of ultrathin sections from vitrified juveniles of *M. galloprovincialis.* **a** Electron-dense granules (arrowheads) were present in the adductor muscle (am) and close to the growing myostracum (my) and nacre (nc). **b** Magnification of the area framed in (**a**), very close to the myostracum, showing the dimensions (red double-headed arrow) of the extrapallial space (eps) and many electron-dense granules (arrowheads). **c** TEM image showing the spatial location of bilayered vesicles (v), ribosome-associated vesicles (rav) and mitochondria (mt) containing electron-dense granules, close to the growing nacre tablets. **d** STEM-HAADF image of a multivesicular body containing bilayered vesicles. **e** Unstained section, showing mitochondrial electron-dense granules close to the growing myostracum. **f** Magnification of the vesicle framed in (**e**), containing various electron-dense granules and some electron-dense diffuse material between them (white arrow). **g** Ribosome-associated vesicle. **h** Mantle cell densely filled with RAVs. **i**. Magnification of the area framed in (**h**) showing the junction (black arrow) between a ribosome-associated vesicle (rav) and a mitochondrion (mt). The white arrow points to some conserved mitochondrial cristae. **j** Mitochondria containing electron-dense granules were observed near the shell (arrow), whereas mitochondria lacking granules were located beneath it (frames). **k** Higher magnification of granule-free mitochondria, framed in (**j**). **l** Mitochondria (arrows) in cells of the inner mantle fold, located away from the shell, lacking electron-dense granules.

The shell of *Mytilus galloprovincialis* is composed of two principal microstructures: an external layer of fibrous prismatic calcite and an internal layer of nacre, both produced by distinct regions of the mantle epithelium. The myostracum, composed of aragonite prisms, is a third microstructure produced by the tendon cells in the adductor muscle attachment zone (Fig. 1a). Mantle cells are virtually in contact with the shell, separated only by a narrow extracellular compartment, known as the extrapallial space. In the adductor muscle attachment zone, it ranged from 70 to 200 nm (Fig. 1b). In this region, tendon cells remain anchored to the shell by collagen fibers that penetrate the myostracum, minimizing dimensional distortion of the extrapallial space during sample preparation^13^.

Through transmission electron microscopy (TEM), three types of organelles were identified near the shell’s growth surface of vitrified juveniles of *M. galloprovincialis*: bilayered vesicles (Fig. 1c–d), ribosome-associated vesicles (Fig. 1c, g–i) and mitochondria containing electron-dense granules (Fig. 1a–c, e–f, h–k). Large clusters of electron-dense mitochondrial granules were also observed along the adductor muscle area (Fig. 1a) and were visible even in unstained sections (Fig. 1e, f). Most mitochondria containing larger electron-dense granules lacked internal cristae (Fig. 1c, e–f), whereas in those with fewer or smaller granules, some cristae were observed (Fig. 1i, white arrow). In cells located away from the shell, such as those in the inner mantle fold, electron-dense granules were absent (Fig. 1j-l).

**Fig. 2:**
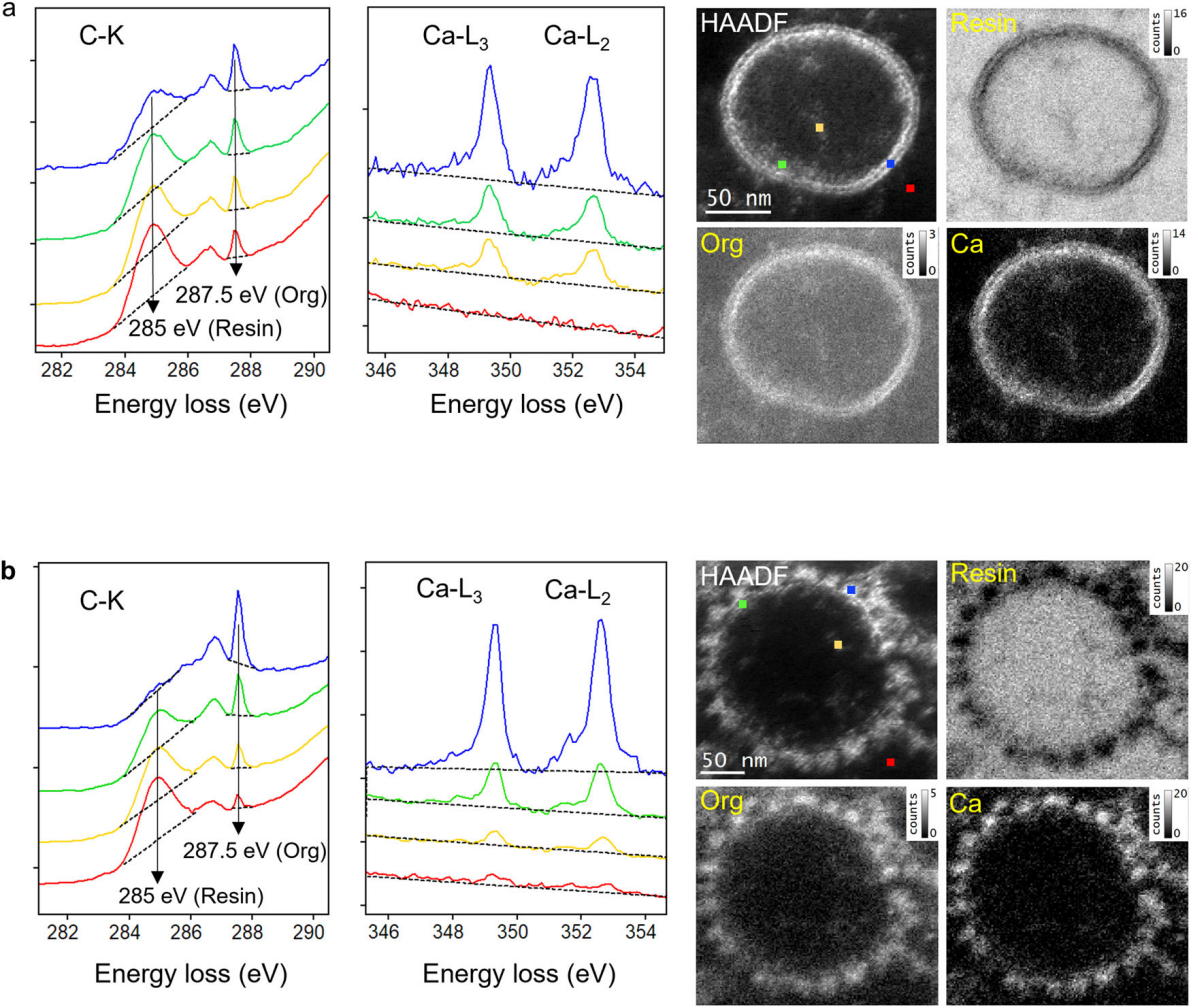
Chemical characterization of bilayered vesicles and RAVs. EEL spectra acquired on a bilayered vesicle (**a**) and a RAV (**b**). Spectra were acquired in the energy range corresponding to carbon (K-edge) and calcium (L_2,3_-edge). Dotted lines show the background subtraction used for each spectral feature to generate the corresponding maps. On the right, HAADF images and spectral maps for resin (285 eV), organic compounds (Org) and calcium (Ca) are shown. The peak at 287.5 eV for carbon edge is characteristic of organic compounds and can be used to map them as reported in previous studies^36,45^.The coloured squares in HAADF images correspond to the areas from which the spectrum of the same colour has been extracted. (Scale, 50 nm, aplicable to all images). A coloured version of spectral maps is presented in Supplementary Fig. 3.

**Fig. 3:**
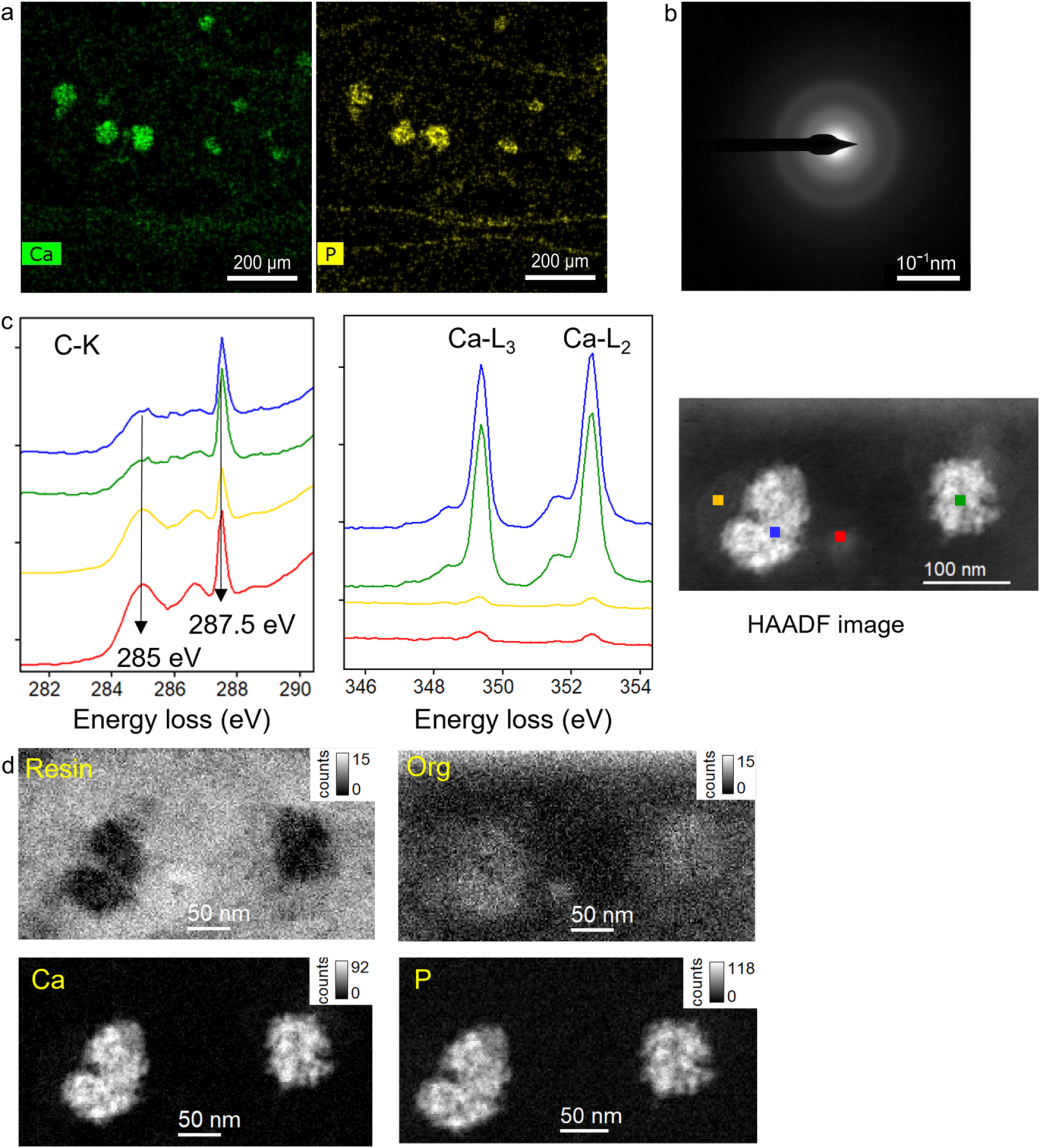
Structure and composition of electron-dense mitochondrial granules. **a** EDX maps for calcium (green) and phosphorus (yellow) revealed a clear signal from various electron-dense granules. (Scale bars, 200 nm). **b** Electron diffraction pattern obtained from the granules, consistent with an amorphous material. **c** EEL spectra in the energy range corresponding to carbon (K-edge) and calcium (L_2_,_3_-edge), obtained from the coloured areas marked in the HAADF image of two granules. The peak at 287.5 eV is characteristic of organic compounds and was used to map their distribution. **d** Maps obtained from EELS data showing the distribution of resin (285 eV), organic compounds (Org), calcium (Ca) and phosphorus (P). A coloured version of spectral maps is presented in Supplementary Fig. 3.

The chemical composition of the three types of organelles was analyzed using monochromated EELS. To achieve this, a scanning transmission electron microscope (STEM) equipped with a state-of-the-art electron monochromator and a direct electron-detector was used, enabling the detection of very weak signals with high spectral resolution. Similar to X-ray absorption spectroscopy measurements, a recent study using this instrument demonstrated the possibility of obtaining phase structure information at 1 nm resolution from the fine structure analysis of the spectral features^36^. Phosphorus and calcium distributions were obtained from their respective atomic edge signals (P-L₂,₃ and Ca-L₂,₃). The carbon spectra exhibited a series of sharp peaks associated with the C 1 s → π* transitions, along with a broad shoulder around 300 eV, characteristic for C 1 s → σ* transitions. The positions of the sharp peaks vary depending on the carbon bonding environment. This near-edge fine structure can be used to analyze the different compounds. The carbonate is detected on the carbon edge by the presence of a peak at 290.3 eV. As in previous EELS studies concerning biominerals^36,45^, the distribution of organic compounds was extracted from the peak at 287.5 eV on the C-K edge (cf. SI Appendix, Supplementary Fig. 1). The phosphorus and calcium maps were obtained from the integration of their corresponding elemental edges after background subtraction, while the different carbon compounds were mapped based on the intensities of their characteristic peaks. Several spectral images were acquired from each type of organelles (Figs. 2, 3d and Supplementary Fig. 2).

### Bilayered vesicles

Bilayered vesicles were round, with an average diameter of 180$\pm$120 nm (n = 50). Some were enclosed within a common membrane, forming multivesicular bodies (Fig. 1d). EELS analyses revealed the presence of calcium, with a signal intensity markedly higher on the bilayer membrane (Fig. 2a, blue curve for Ca-L₂,₃ edge) and a slightly lower signal associated with a diffuse cargo (Fig. 2a, green and orange curves for Ca-L₂,₃ edge), which was predominantly bound to the luminal side of the membrane (Fig. 2a, green square in the organic map). As expected, the bilayer membrane was associated with a higher intensity of the organic compound peak at 287.5 eV (blue curve for C-K edge in Fig. 2a), and was clearly visible on the corresponding map.

Calcium-loaded vesicles related to gastropod nacre formation have been recently studied by our group^14^. In that study, numerous bilayered vesicles were found close or attached to the mantle side of the surface membrane, which closes the nacre growth compartment. Elemental analyses with EELS and EDX showed that vesicles transported calcium associated with organic matter, preferentially bound to the inner face of the bilayer membrane^14^. The bilayered vesicles described in the present study (Figs. 1c–d and 2a) exhibit similar size, structural features, cargo distribution, and composition as those previously described^14^. However, the calcium signal intensities obtained in the present study are markedly higher. This is likely due to the cryofixation techniques employed (high-pressure freezing, HPF, followed by freeze substitution, FS), which provide substantially better preservation of native conditions than the freeze-drying method used by Macías-Sánchez et al.^14^ in preserving native conditions. Additionally, the use of monochromated STEM–EELS provides improved spectral resolution, and a higher signal-to-noise ratio thanks to the Timepix3 direct electron detector enables mapping of different compound distributions at nanometric resolution. Despite these differences, there is a clear correspondence between the vesicles identified in this study and those observed on the surface membrane of gastropods, which are clearly associated with nacre mineralization^14^. Therefore, it is reasonable to assume that these bilayered vesicles containing calcium associated with organic compounds also contribute to the mineralization process in bivalves.

Based on their size (~50–300 nm) and their localization within multivesicular bodies (Fig. 1d), bilayered vesicles may be tentatively classified as exosomes^49,50^. Notably, many matrix vesicles, commonly defined in biomineralization contexts as calcifying vesicles present in extracellular matrices, have been proposed to be exosomes^51,52^. However, confirmation of their exosomal identity would require additional evidence, including the detection of specific protein markers^50,52^.

### Ribosome-associated vesicles (RAVs)

RAVs are highly dynamic subcompartments of the endoplasmic reticulum (ER), recently described by Carter et al.^53^. They are usually found at the periphery of secretory cells, where they sustain the protein demand and provide mobile calcium stores involved in local calcium signalling^53^.

Some mantle cells of *M. galloprovincialis* contained numerous RAVs (Fig. 1c, g–h), which were rounded, or occasionally oval, with an average diameter of 320$\pm$140 nm (n = 50) and a bilayer membrane covered with 15–20 nm ribosomes (Fig. 1c, g-i). Calcium and organic signal intensities were higher in the ribosomes (Fig. 2b, blue curves) and lower in the diffuse cargo (Fig. 2b, green and orange curves), which preferentially bound to the luminal side of the membrane (Fig. 2b, green square position in the organic map). A correlation between the intensities of the organic and calcium peaks was observed across the different positions in both bilayered vesicles and RAVs (carbon and calcium curves in Fig. 2a and b), suggesting that calcium and organic compounds were associated.

The relatively low calcium concentration in RAV cargo may be unexpected, given that the ER is generally recognized as the principal intracellular calcium store^54^. However, under calcium overload stress, sustained or elevated calcium fluxes can be triggered from the ER to mitochondria. Mitochondria are capable of accumulating substantial amounts of calcium within their matrix, thereby buffering cytosolic and ER calcium overload^55–57^. The presence of Ca- and P-rich granules in the mitochondrial matrices (Fig. 1a–c, e–f) provides evidence of calcium accumulation within mitochondria. This calcium is likely derived from RAVs, which were occasionally observed in direct contact with mitochondria (Fig. 1i). These connections occur through specific membrane contact sites, known as mitochondria–ER contacts (MERCs) or mitochondria-associated membranes (MAMs). These contact sites facilitate the transfer of lipids and calcium from the ER to the mitochondria, which is key for lipid synthesis and for maintaining calcium signaling and homeostasis^53,54^.

### Electron-dense mitochondrial granules

Electron-dense mitochondrial granules exhibited irregular rounded shapes and mean diameters of 70 ± 20 nm (n = 50), with the largest ones measuring approximately 100 nm (Figs. 2f and 3d). Frequently, various granules were spatially clustered (Fig. 1b–c, f), and some diffuse electron-dense material appeared between them (Fig. 1f, white arrow). Energy-dispersive X-ray spectroscopy (EDX) revealed calcium and phosphorus as the predominant elements (Fig. 3a). An average, calibrated Ca/P ratio of 1.3 ± 0.2 (with individual values ranging from 1.1 to 1.7; n = 35) was obtained using inorganic hydroxyapatite as a standard. No diffraction pattern was observed (Fig. 3b), indicating that the granules were amorphous in nature. EELS confirmed a high content of phosphorus and calcium, in addition to the presence of organic compounds (Fig. 3c–d). Moreover, the distributions of phosphorus and calcium appeared similar, as indicated by matching signal intensity maps for both elements in EDX and EELS data (Fig. 3a and d). EELS analyses of electron-dense granules were performed on both stained and unstained sections (Supplementary Fig. 4), with no differences observed between the EEL spectra obtained under either condition. The small peak in the carbon K-edge around 290.9 eV (Fig. 3c) is consistent with the presence of proteins (cf. SI Appendix; Supplementary Fig. 1).

**Fig. 4:**
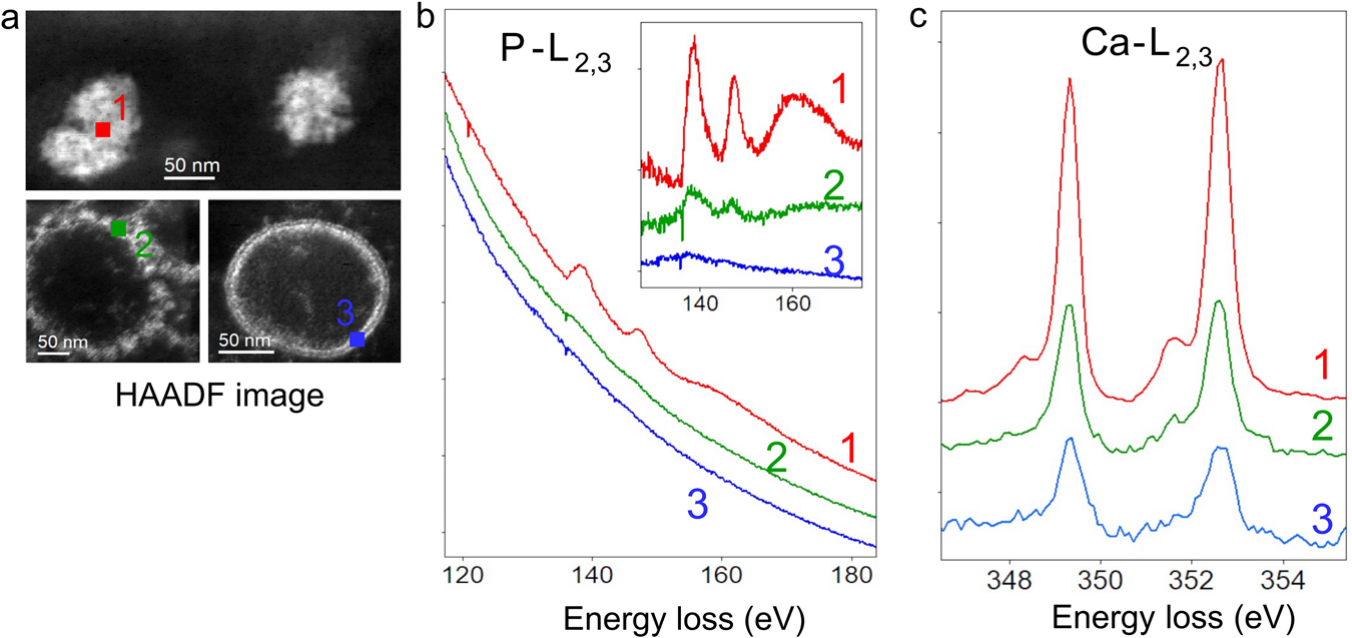
Comparison of phosphorus and calcium signals from the different organelles. **a** HAADF images showing the locations of the analyzed areas (electron-dense granule (1), RAV (2), and bilayered vesicle (3)). **b**, **c** EEL spectra extracted from the areas marked in the HAADF image: **b** in the energy range encompassing the phosphorus L_2_,_3_-edges. The presented spectra were summed over a 6×6 px² area. The background-subtracted spectra are shown in the inset; **c** in the energy range corresponding to calcium L_2_,_3_-edges.

The phosphorus content in the mitochondrial granules was markedly higher than that detected in the ribosomes of RAVs and in the plasma membrane of bilayered vesicles (Fig. 4b). In the latter, despite its phospholipid composition, the phosphorus signal was not visible, likely because it was below the detection limit under the current experimental conditions (Fig. 4b, blue curve). The calcium signal was also markedly higher in the granules compared to the other organelles (Fig. 4c). The overall calcium pattern in the granules (Fig. 4c, red curve) was very similar to that observed for synthetic and biogenic calcium phosphates^26,58,59^. The positions of the main L₂ and L₃ peaks have been reported to be very constant for all calcium phosphate compounds, whereas variations in the number, positions, and intensities are observed for the smaller pre-edge peaks. Here, the two most intense minor peaks were shifted by approximately 1.1 eV from the L₂ line and 1 eV from the L_3_ line.

CaP granules have been proposed to consist of either amorphous calcium phosphate^60^ or amorphous calcium polyphosphate^61^, although their exact composition remains uncertain. In bone tissue, CaP granules have been observed to undergo a maturation process. They initially appear as amorphous structures with a low Ca/P ratio within the mitochondria of osteoblasts, and progressively undergo crystallization. This is accompanied by an increase in Ca/P ratio through a series of intermediate phases, ultimately giving rise to hydroxyapatite in the extracellular matrix^40,45^. In our analysis, the Ca/P ratio was approximately 1.3 ± 0.2, which corresponds with values reported in osteoblast mitochondria^45^ as well as values proposed for amorphous calcium phosphate (ACP) and octacalcium phosphate (OCP)^62,63^. Electron diffraction indicated that the granules were amorphous in nature (Fig. 3b), which is consistent with the presence of ACP. Concerning EELS data, the positions of the minor peaks (Fig. 4b) are shifted by approximately 1 eV relative to the main lines. To the best of our knowledge, only one study^26^ has provided spectral information on the Ca L₂,₃-edge in ACP, reporting a 0.5 eV splitting of the minor peak from the main L₃ line. In the present study, the value (~1 eV) measured on the granules corresponds to those associated with crystalline phases in Beniash et al.^26^, which contrasts with the amorphous nature suggested by the Ca/P ratio and the electron diffraction pattern. This discrepancy may be attributed to beam-induced recrystallization during EELS measurements, although this phenomenon has not been reported in previous EELS analyses of amorphous calcium carbonate^36^. Moreover, the presence of organic compounds suggests the existence of a mineral-organic mixed phase. Some of these organic molecules may correspond to extracellular matrix proteins or proteins involved in mineralization processes, as has been proposed in the context of bone formation^45^. Additionally, other molecules such as ATP could be present, which may contribute to the stabilization of the granules, as well as to the phosphorus content^40^.

Although carbonate may also accumulate in mitochondria^64^, we did not detect any carbonate signal in CaP granules. However, given the current spectral resolution (~ 0.1 eV), the peak widths, and the proximity of the 290.9 eV peak to the carbonate peak at 290.3 eV, the presence of trace amounts of carbonate cannot be ruled out. Nonetheless, we did not find evidence of carbonate presence in other studies on mitochondrial granules. Nitiputri et al.^45^ detected carbonate in the core of very similar granules, though these were found in the extracellular matrix of bone.

### CaP granules as conserved calcium storages

Our present study shows that mitochondrial CaP granules in mollusc mantle cells are equivalent to those found in bone-forming cells^40,44,47^. Despite their widespread occurrence in bone tissue, mitochondrial electron-dense granules have been scarcely reported in organisms producing calcium carbonate skeletons. They have been found in (a) the calciferous glands of the common earthworm *Lumbricus terrestris*, where they are implicated in calcite production for calcium elimination^65^; (b) mitochondria isolated from the hepatopancreas of the blue crab *Callinectes sapidus*, where they are proposed to serve as temporary calcium storage during moulting^66^; and (c) cultured primary mesenchyme cells of the sea urchin embryo^67^. However, these studies did not resolve the composition or structural characteristics of the granules. Chen et al.^66^ reasonably assumed that the granules were composed of calcium and phosphate due to the significant accumulation of both elements in the mitochondria after culture. They also found that some granules consisted of bundles of needle-like crystals^66^. These bundles likely originated from traditional chemical fixation procedures. This artifact could be prevented by cryofixation methods, such as high-pressure freezing (HPF), which was used in this study to better preserve the native structure of the granules^40,44,47^.

In contrast, the formation of mitochondrial CaP granules has long been described in bone-related cells^16,39,41–44,68–70^ and in non-calcifying tissues or isolated mitochondria under pathological or experimental conditions involving high calcium input^71–76^. Their extensive occurrence in different cell types highlights their physiological significance in buffering elevated intracellular and intramitochondrial calcium concentrations. Although calcium was initially considered toxic to mitochondria, subsequent studies demonstrated that low concentrations of calcium stimulate respiration. Moreover, calcium accumulation within mitochondria may take precedence over oxidative phosphorylation, highlighting its fundamental role in metabolism^60^. Nevertheless, as mentioned above, calcium overload within the mitochondrial matrix leads to the formation of amorphous electron-dense granules enriched in calcium and phosphorus, typically adjacent to cristae^60,71,72^. These CaP granules contribute to maintaining a constant free calcium concentration within the mitochondrial matrix, which is essential for preserving mitochondrial function. However, sustained calcium overload can compromise mitochondrial metabolism and potentially trigger the mitochondrial permeability transition (MPT). This event may subsequently lead to mitophagy (the selective autophagic degradation of mitochondria), and ultimately result in apoptosis or necrosis^46,77,78^.

Due to the high amounts of calcium and phosphorus sequestered in CaP granules, their involvement in mineralization was proposed^60,79^. Their presence in a wide range of cell types does not preclude this possibility. In fact, mineralization is a ubiquitous process that can occur even in non-mineralizing tissues upon injury^60^. Therefore, the uniqueness of mineralizing tissues likely resides in their ability to control the mineral ion pathways, either through specific organic molecules or the specific regulation of the organelles involved, as well as in the production of extracellular organic matrices that serve as scaffolds for crystallization^48,60,64,79–81^.

Indirect evidence for the involvement of mitochondria in mineralization was provided by Ianotti et al.^82^, who found that mitochondria from isolated growth-plate and hyaline-cartilage chondrocytes exhibited higher endogenous calcium content, an increased capacity for calcium accumulation, and a larger labile calcium pool as compared to the mitochondria of hepatocytes. More recent studies also supported a precursor role for CaP granules in bone formation, proposing two distinct pathways for their transport and release into the extracellular matrix: either via prior transfer to intracellular vesicles^44^ or through mitophagy^46^. The latter is consistent with additional findings demonstrating a positive correlation between autophagy and mineralization^83–85^. In the context of the former pathway, mitochondria may have been misidentified as other vesicles, since calcium precipitates can disrupt mitochondrial morphology, leading to the loss of their characteristic inner membrane cristae (Fig. 1f)^76,78^.

There is increasing evidence that phosphate plays a key role in the regulation and deposition of calcium carbonate^37^. Mixed phases of calcium, phosphate, and carbonate have recently been observed during the early mineralization stages of the abalone shell, suggesting the existence of a conserved mineral precursor^48^. This finding reinforces the idea that biomineralization is influenced by ancestral regulatory mechanisms of intracellular calcium^86^. Similar suggestions have linked the acidocalcisomes of non-mineralizing algae with calcium polyphosphate bodies found in coccolithophores^87,88^. It has been proposed that, in addition to playing a key role in the regulation of intracellular calcium, these CaP pools may also be involved in the mineralization of coccolith calcite^88^.

The presence of mitochondrial CaP granules in organisms that produce calcium carbonate further supports the idea that ancestral calcium pathways are involved in both the regulation of intracellular calcium metabolism and biomineralization. However, the mechanisms by which calcium concentrated in CaP granules is mobilized and transported to the shell remain to be elucidated. Similar to bone, mitophagy could be involved^46^. Subsequently, specific proteins and organic matrices^79,81^, along with the chemistry of the mineralization compartment, are likely critical in the regulation and transformation toward the final mineral phases.

**Fig. 5:**
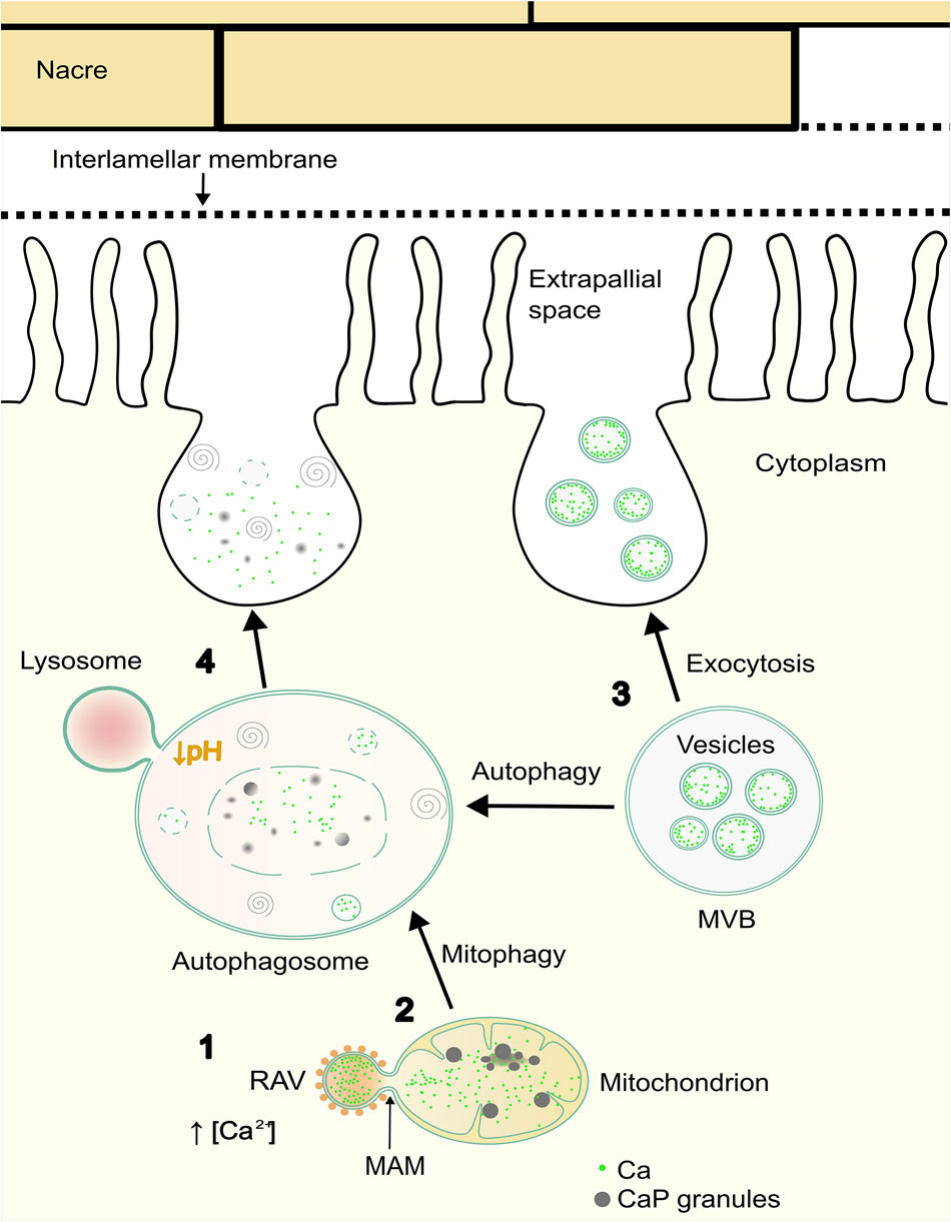
Proposed schematic model of intracellular calcium trafficking in mantle cells. (**1**). An increase in cytoplasmic calcium concentration triggers the transfer of calcium from the endoplasmic reticulum or ribosome-associated vesicles (RAV) to the mitochondria via mitochondrion-associated membranes (MAMs). (**2**). Calcium overload in the mitochondrial matrix leads to the formation of CaP granules. This sustained condition activates mitophagy, which results in the formation of an autophagosome. (**3**). Multivesicular bodies release bilayered vesicles containing calcium associated with organic species directly into the extrapallial space by exocytosis or, alternatively, direct them to the autophagic pathway. (**4**). Lysosomes release their acidic hydrolases into the autophagosome, forming an autophagolysosome where organelle digestion occurs. Due to the acidic environment of the autophagolysosome (pH ~5), CaP granules may dissolve into ions or smaller particles. Once released into the extrapallial space, these products could contribute to the mineralization process.

The findings of this article, together with existing knowledge from other studies, lead us to propose the model illustrated in Fig. 5. Initially, an increase in intracellular calcium concentration triggers the redirection of calcium from the ER or RAVs toward the mitochondria, where it becomes concentrated and stored in CaP granules. Upon initiation of mitophagy, these granules may dissolve due to the acidic environment (pH ~5) generated by lysosomal hydrolases following autophagosome-lysosome fusion, resulting in the formation of an autophagolysosome. The products of mitophagy are subsequently released into the extrapallial space via exocytosis. Simultaneously, bilayered vesicles contained within multivesicular bodies may be released into the extrapallial space, where they may either release their calcium content or be redirected to the autophagic pathway. We hypothesize that the calcium concentrated within the granules must first be converted into smaller particles or dissolved into ions to traverse the pores of pre-assembled interlamellar organic membranes of nacre. During this process, the calcium may also become enriched in carbonate. Further evidence on calcium transport from CaP granules to the shell, and its transformation into the final precursor phase, is needed to support this hypothesis and complete the framework of this “highway to shell”.

## Materials and methods

### Specimen preparation by High-pressure freezing (HPF) and Freeze-substitution (FS)

To preserve the native conditions of the samples as closely as possible, the specimens were vitrified using cryofixation by HPF, followed by FS of water. This prevents the structural and compositional changes typically induced by chemical fixatives, as well as the crystallization of amorphous phases caused by aqueous solutions^40,47^.

Six juvenile *Mytilus galloprovincialis* (exhibiting adult morphology but still sexually immature), approximately 1 mm in width, were collected in July 2024 from intertidal rocks in the littoral of Málaga (36° 34′ 48″ N, 4° 33′ 13″ W), southern Spain. In the laboratory, both valves of fresh specimens were cut apart under a stereo microscope and deposited in HPF planchets immersed in 20% BSA. Subsequently, the samples were vitrified by HPF using a Leica EM HPM100.

Right after HPF, the planchets were transferred in liquid nitrogen to a Leica EM AFS2 freeze substitution processor where each sample was immersed in a vial containing an anhydrous acetone solution mixed either with (a) 1% glutaraldehyde; (b) 0,1% uranyl acetate or (c) 0,1% uranyl acetate and 0,1% osmium tetroxide. The vials were kept at -90°C for 48 h. The temperature was then gradually raised by 5°C/h to -20°C and maintained at that temperature for 12 h. Finally, the temperature was increased to 4°C at a rate of 5°C/h.

### Transmission electron microscopy (TEM)

The samples were embedded in Spurr low viscosity embedding resin (EMS). Ultrathin sections of 100 nm were obtained with an ultramicrotome Leica EM UC7/FC7 and collected in carbon supported copper grids for their observation in a JEOL-JEM1400 to obtain TEM bright field-images.

### Scanning transmission electron microscopy (STEM)–energy-dispersive X-ray spectroscopy (EDX)

Ultrathin sections of 100 nm obtained with an ultramicrotome Leica EM UC7/FC7 were observed in a FEI Talos F200X, equipped with 4 in-column SDD Super-X detectors. High-angle annular dark-field (HAADF) STEM images were obtained, and the elemental composition of the electron dense granules was analyzed by EDX. Semiquantitative data obtained from EDX spectra were used to calculate Ca/P ratios, calibrated using inorganic hydroxyapatite as a standard.

### Electron energy loss spectroscopy (EELS) acquisition and processing

Ultrathin sections of 50 nm obtained with an ultramicrotome Leica EM UC7/FC7 were collected on copper grids with lacey carbon films for EELS analyses. EELS data were acquired using a Cs-corrected Nion Hermes 200-S microscope operated at 100 kV. This system is equipped with a gun monochromator, a Nion Iris spectrometer, a single-tilt cryo-specimen holder (LN₂ cooling holder from HennyZ) to minimize beam-induced damage, and a CheeTah Timepix3 camera commercialized by Amsterdam Scientific Instruments. Although the CheeTah is an event-based detector, it was used in this study in the frame-based mode. Spectrum-imaging was performed by raster-scanning the electron beam over the area of interest with a pixel size of 1 nm, a dwell time between 1 and 3 ms, and a beam current of 25 pA resulting in electron doses of 4000 e^-^/Å^2^. At each pixel, a complete EEL spectrum was simultaneously acquired alongside a HAADF image. Typically, a hyperspectral image consisted of approximately 50,000 spectra. Spectra were acquired with an energy dispersion of ΔE = 0.1 eV/channel over the ranges of 255–360 eV (covering the carbon K-edge and calcium L₂,₃-edges) and 90–195 eV (encompassing the phosphorus L₂,₃-edges).

Energy scale calibration was based on reference values reported in the literature^36,59^, using the carbonate peak at 290.3 eV, the calcium L_2_-edge peak at 349.3 eV for the energy range 255–360 eV, and the phosphorus L₂, ₃-edge peaks at 138.5 eV and 147 eV for the energy range 90–195 eV, as described in our previous study^36^. The use of a monochromated electron beam enabled high energy resolution, reaching 150 meV in the core-loss region. Data was binned, denoised using principal component analysis (PCA) implemented in Gatan’s Digital Micrograph software, and gain corrected. After background subtraction, elemental maps were generated by integrating the intensity over the corresponding spectral signatures: the L₂, ₃-edge double peaks indicated above for calcium and phosphorous, and the peak at 287.5 eV of the carbon K-edge for organic compounds (cf. SI Appendix; Supplementary Fig. 1).

### Reporting summary

Further information on research design is available in the Nature Portfolio Reporting Summary linked to this article.

## Supplementary information

Transparent Peer Review file

Supplementary Information

Reporting Summary

## Data Availability

All data needed to evaluate the conclusions are present in the manuscript and the Supplementary Materials. All raw data are available from the corresponding authors upon reasonable request.
