# Supplementary material for: Calcium trafficking in the mussel Mytilus galloprovincialis suggests conserved biomineralization pathways
Source: Commun Biol. 2026 May 11;9:814. doi: 10.1038/s42003-026-10210-2 (PMC13269901; doi:10.1038/s42003-026-10210-2)
Supplement: Supplementary file 2 — Supplementary Information [file 42003_2026_10210_MOESM2_ESM.pdf]

Supporting Information

Calcium trafficking in the mussel *Mytilus galloprovincialis* suggests conserved biomineralization pathways

Authors and Affiliations

Ernesto Ruiz-Villaespesa[1]*, Antonio G. Checa[1], Xiaoyan Li[2], Carmen Salas[3]*, Marta de Frutos[2].

[1]Departamento de Estratigrafía y Paleontología, Facultad de Ciencias, Universidad de Granada, 18071 Granada, Spain.

[2]Laboratoire de Physique des Solides (LPS), CNRS UMR 8502, Université Paris-Saclay, F-91405 Orsay, France.

[3]Departamento de Biología Animal, Facultad de Ciencias, Universidad de Málaga, 29071 Málaga Spain.

***Corresponding authors**:

Ernesto Ruiz-Villaespesa (rvillaespesa@correo.ugr.es)

Carmen Salas (casanova@uma.es)

Methods-Electron Energy Loss Spectroscopy (EELS) Acquisition and Processing

EELS analysis of carbon K-edge

The spectra exhibited a series of sharp peaks associated with the C 1s → π* transitions, along with a broad shoulder around 300 eV, characteristic for C 1s → σ* transitions. The positions of the sharp peaks vary depending on the carbon bonding environment. The carbonate peak at 290.3 eV from the aragonite tablets was used as a reference to calibrate the energy scale according to Cosmidis et al.[1]. The spectrum recorded from the mitochondrial granule (blue curve in Fig. S1A) reveals four characteristic peaks at 285 eV, 286.5 eV, 287.5 eV, and 290.9 eV. Given the electron doses applied in this study (≈4000 $e^-/Å^2$), these spectral features can be attributed to the degradation of chemical compounds induced by electron beam irradiation. The peaks at 285 eV and 286.5 eV have been assigned to 1s → π* transitions in C=C and C≡C bonds, respectively. For instance, the peak at 285 eV is observed for amorphous carbon and embedding resin. The main peak at 287.5 eV was used to map organic compounds in previous EELS studies concerning biominerals[2–4]. In early studies, this peak was attributed to the 1s–π* C=O

transition in carbonyl groups but recent studies have suggested that this very narrow peak is associated with carbon monoxide produced by the radiolysis of certain organic compounds[5,6]. Similarly, the peak at 290.9 eV has been attributed to the carbon dioxide generated from organic compounds (for instance proteins or DNA)[5,6]. The EELS maps obtained for the mitochondrial granule by integrating the different carbon peaks are presented in Fig. S1C. Consistent with their respective assignments, the distributions associated with the peaks at 285 eV and 286.5 eV exhibit maximum intensity outside the calcium phosphate granule, in regions of higher resin concentration, whereas those at 287.5 eV and 290.9 eV show maximum intensity within the granule.

In the present study, the main peak at 287.5 eV was chosen to map organic compounds in the specimen, as its strong signal ensures better accuracy compared with the nitrogen K-edge, which is considerably noisier than the carbon one due to the low intensity of N signal[7]. Throughout the text, we refer to the presence of this peak as indicative of organic compounds, most likely represented by proteins.

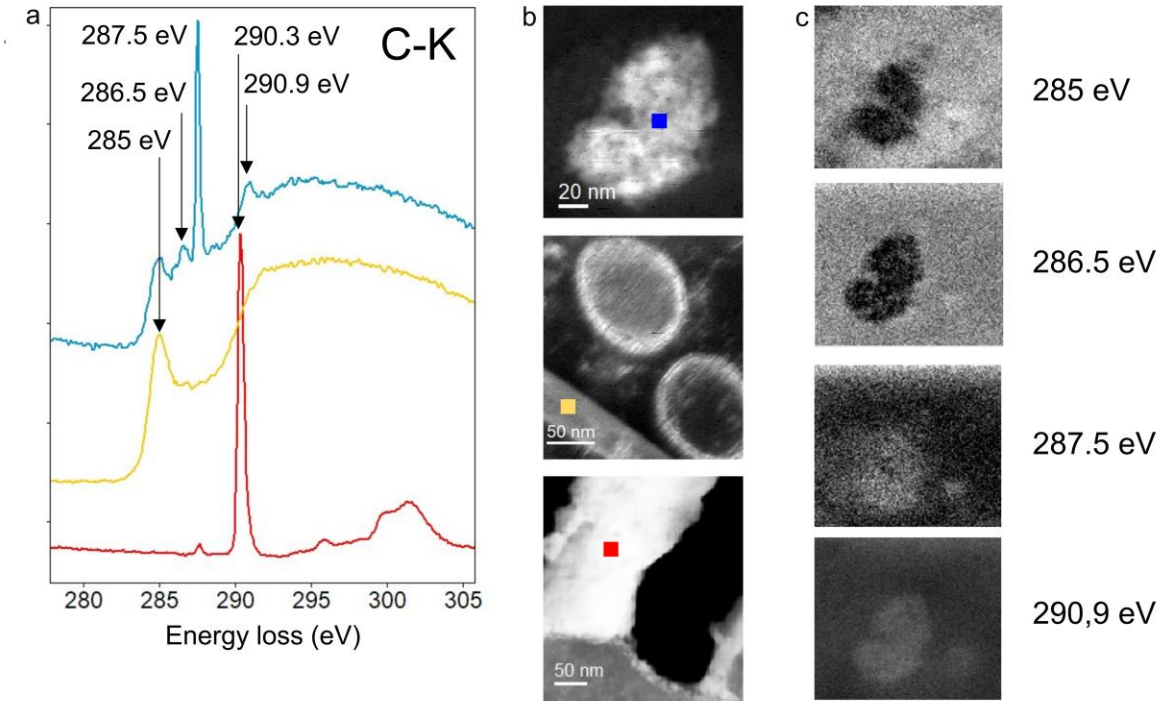

Supplementary Figure 1. A. Carbon K-edge EEL spectra from a mitochondrial granule (blue curve), the amorphous carbon from the TEM grid film (yellow curve), and an aragonite tablet (red curve), from the positions labeled on the HAADF images (**B**). **C.** EELS maps obtained by integrating the different C K-edge peaks from the mitochondrial granule (top image in B).

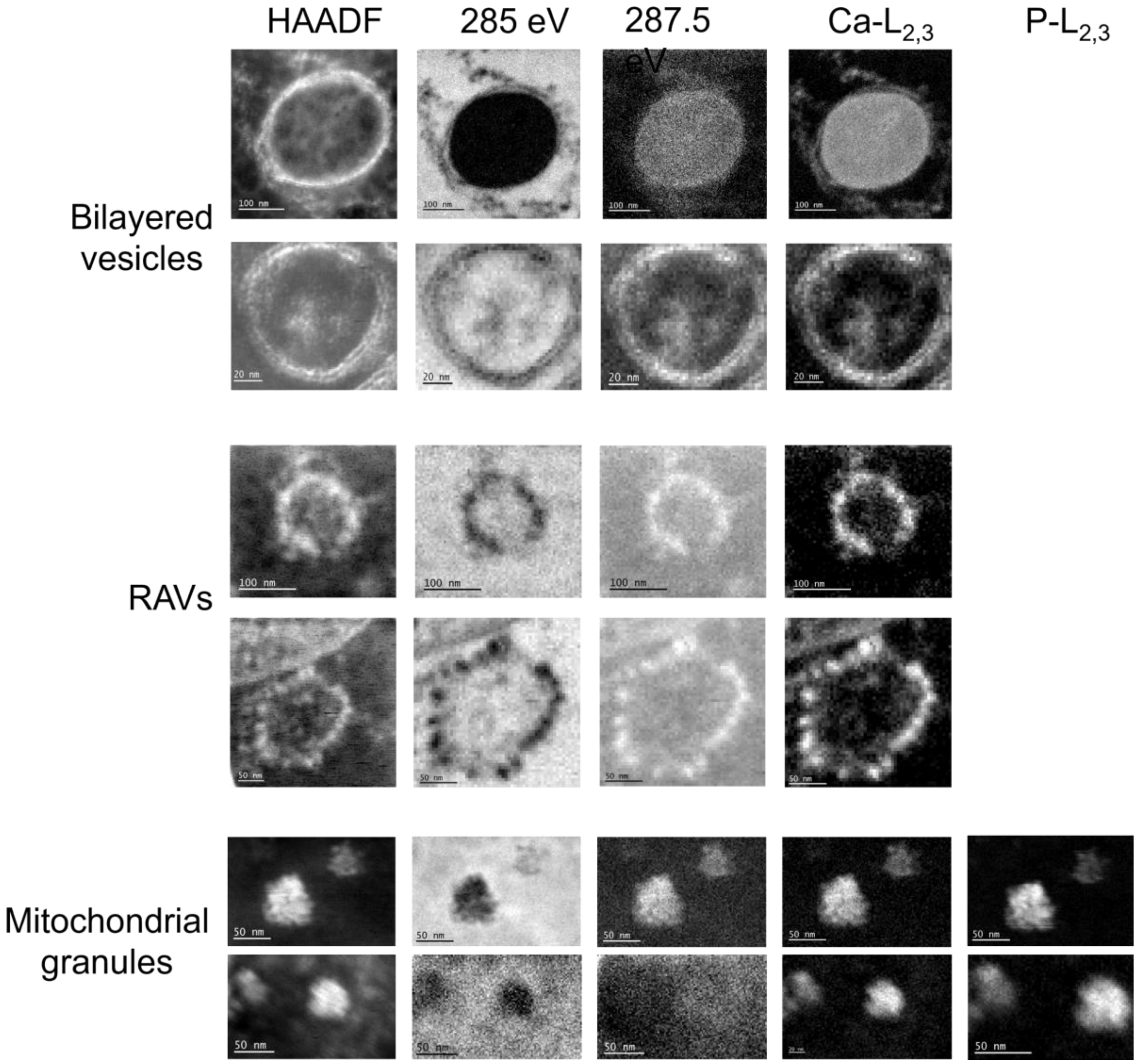

Supplementary Figure 2. HAADF and chemical maps obtained from the peak signals at 285 eV (resin), 287.5 eV (Org), 349.3 eV–352.6 eV (Ca-$L_{2,3}$) and 138.5 eV–147 eV (P-$L_{2,3}$), within bilayered vesicles, RAVs and mitochondrial granules.

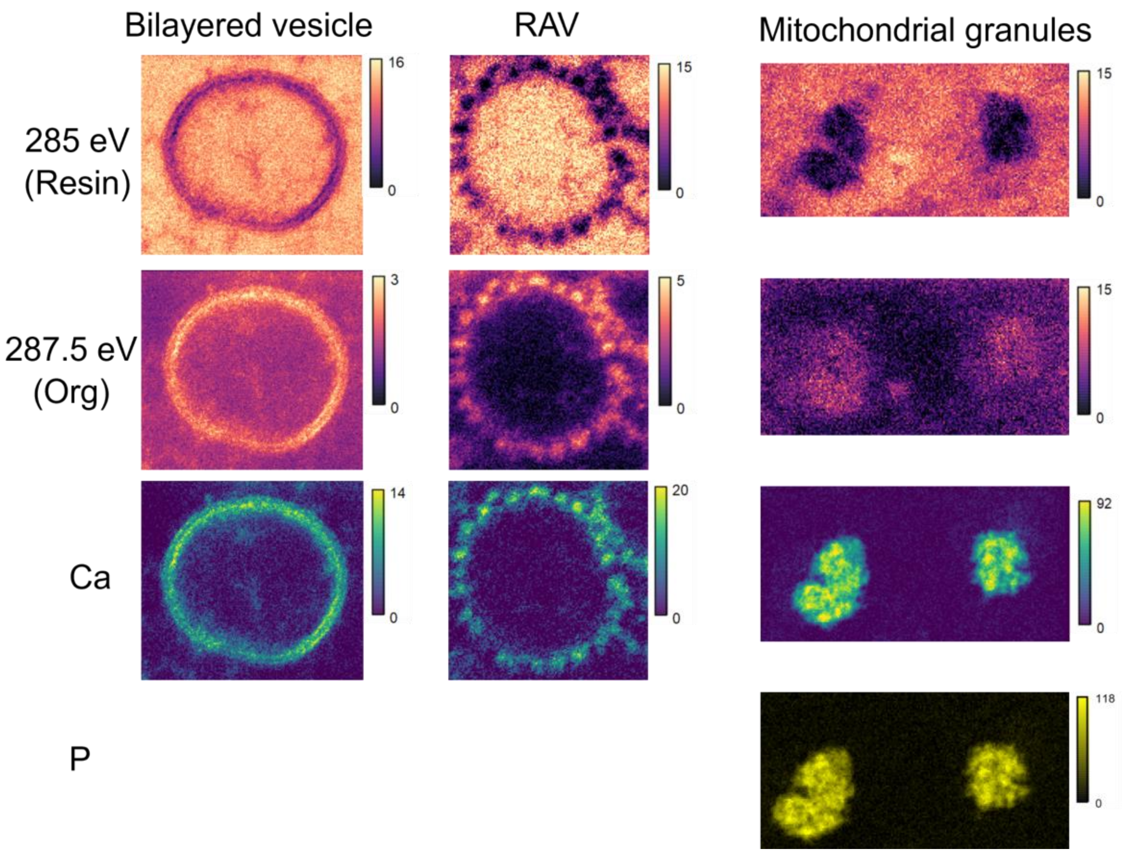

Supplementary Figure 3. Coloured spectral maps corresponding to those of the Figure 2 and Figure 3d, acquired in the energy ranges corresponding to carbon (K-edge), calcium ($L_{2,3}$-edge) and phosphorous ($L_{2,3}$-edge).

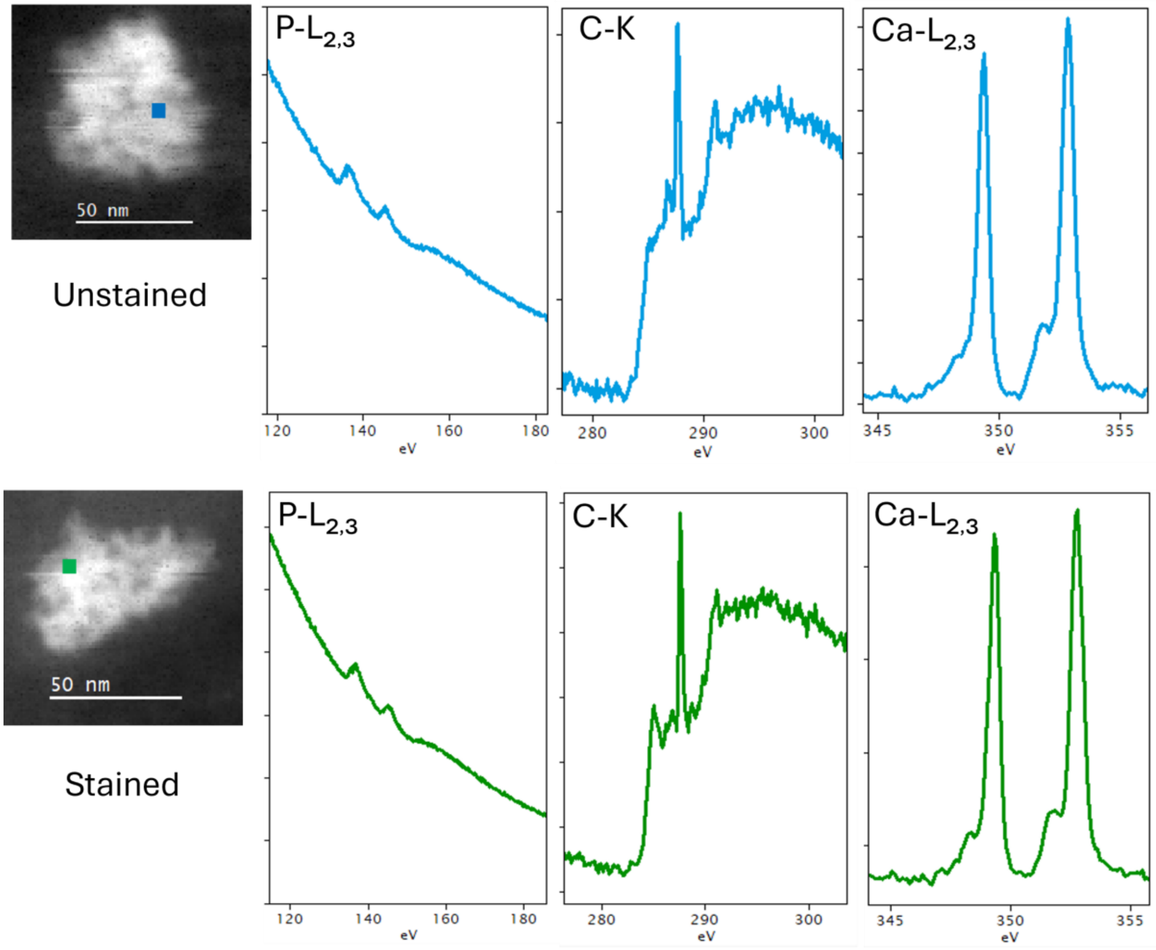

Supplementary Figure 4. EEL spectra acquired in the energy ranges corresponding to phosphorous ($L_{2,3}$-edge), carbon (K-edge) and calcium ($L_{2,3}$-edge) from both unstained and stained electron-dense granules.

Supplementary References

1. Cosmidis, J., Benzerara, K., Nassif, N., Tyliszczak, T. & Bourdelle, F. Characterization of Ca-phosphate biological materials by scanning transmission X-ray microscopy (STXM) at the Ca L2,3-, P L2,3- And C K-edges. *Acta Biomater* **12**, 260–269 (2015).

2. Nitiputri, K. *et al.* Nanoanalytical electron microscopy reveals a sequential mineralization process involving carbonate-containing amorphous precursors. *ACS Nano* **10**, 6826–6835 (2016).

3. de Frutos, M., Rodríguez-Navarro, A. B., Li, X. & Checa, A. G. Nanoscale analysis of the structure and composition of biogenic calcite reveals the biomineral growth pattern. *ACS Nano* **17**, 2829–2839 (2023).

4. Kłosowski, M. M. *et al.* Electron microscopy reveals structural and chemical changes at the nanometer scale in the osteogenesis imperfecta murine pathology. *ACS Biomater Sci Eng* **3**, 2788–2797 (2017).

5. Colby, R. *et al.* Identifying and imaging polymer functionality at high spatial resolution with core-loss EELS. *Ultramicroscopy* **246**, 113688 (2023).

6. Chaupard, M. Electron spectromicroscopy: from organic-inorganic drug nanocarriers to biological systems. (2023) doi:10.34894/VQ1DJA.

7. Gay, C. et al. Nanoscale Analysis of Randall's Plaques by Electron Energy Loss Spectromicroscopy: Insight in Early Biomineral Formation in Human Kidney. ACS Nano **14**, 1823–1836 (2020).
