# Supplementary material for: Calcium trafficking in the mussel Mytilus galloprovincialis suggests conserved biomineralization pathways
Source: Commun Biol. 2026 May 11;9:814. doi: 10.1038/s42003-026-10210-2 (PMC13269901; doi:10.1038/s42003-026-10210-2)
Supplement: Supplementary file 1 — Transparent Peer Review file [file 42003_2026_10210_MOESM1_ESM.pdf]

nature portfolio

Peer Review File

Calcium trafficking in the mussel Mytilus galloprovincialis suggests conserved biomineralization pathways

Corresponding Author: Mr Ernesto Ruiz-Villaespesa

This file contains all reviewer reports in order by version, followed by all author rebuttals in order by version.

Attachments originally included by the reviewers as part of their assessment can be found at the end of this file.

Version 0:

Reviewer comments:

Reviewer #1

(Remarks to the Author)
This is a very interesting manuscript, with beautifully performed experiments followed by thoughtful interpretation.
The authors applied advanced electron microscope techniques to examine the structure and composition of vesicles in cells directly involved in building the shell of a mollusk bivalve. To achieve their purpose, they used a combination of advanced electron microscopy techniques on samples prepared such that they cause minimal change, allowing the observation of samples in close to native conditions. Three vesicle types were analyzed, including bilayered vesicles, ribosome associated vesicles and mitochondria. The mitochondria contained calcium phosphate granules, and this evidence "together with the increasing evidence for the involvement of phosphate in regulating calcium carbonate-based systems, leads us to propose the existence of conserved biomineralization pathways from invertebrates to vertebrates, rooted in an ancestral mechanism of intracellular calcium regulation" (citation from the manuscript). This is a most important statement, supported by the first evidence of the presence of Ca/P containing mitochondrial granules in the shell building cells of mollusks. For this reason, together with the high quality of the work, I recommend acceptance of the paper in COMMSBIO.
The authors propose a complex model involving high cytoplasmic calcium concentration triggering formation of Ca/P mitochondrial granules, followed by mitophagy, autophagolysosome formation, and exocytosis, together with other parallel alternative pathways. As pointed out by the authors, with due caution, "Further evidence on calcium transport from CaP granules to the shell, and its transformation into the final precursor phase, is needed to support this hypothesis". This is especially true in the light of other studies that recorded phosphorus involvement in vesicles in sea urchin embryos (ref 37 in the manuscript), side by side with calcium extraction and concentration from sea water endocytosis. When, how and if a shift from calcium phosphate to carbonate occurs is the real mystery.
Specific comments follow:
Page 4:
- 1mm size animals were selected. Please clarify if these are adults, veligers or larvae. This is important, because in larvae, the shell is known to be composed of amorphous calcium carbonate, and the shell deposition mechanism may be different. If the experiments were performed on larvae or veligers, do you believe that the conclusions of your work are valid also for adults?
- A statement is made, that "Mantle cells are virtually in contact with the shell, separated only by a narrow extracellular compartment, known as the extrapallial space", and this is indeed what is observed, but I wonder whether this space may readjust from farther away to close contact depending on whether, where and when the shell mineral is being deposited at the time of sample preparation.
Page 8:
"A correlation between the intensities of the organic and calcium peaks was observed across the 162 different positions in both bilayered vesicles and RAVs (carbon and calcium curves in Fig. 2A and 2B)". I agree that there is a clear correlation between the curves in figure 2A, but I cannot see a good correlation in figure 2B. Please reconsider and explain

Reviewer #2

(Remarks to the Author)
The paper addresses the role of different organelles in mollusc shells mineralization, using S/TEM and EELS to

demonstrate the presence of minerals in the organelles and propose a biomineralization pathway. While biomineralization pathways are of clear importance across a variety of organisms and the methodology used is interesting, I find certain aspects to be of great concern in the current form of the manuscript. In my opinion, these concerns are too fundamental to be addressed through revision, and therefore I cannot recommend this manuscript for publication.

My biggest concern regards Figure 2: it seems quite apparent to me that the EELS maps for the organics and calcium are indeed the same map with different brightness and contrast. This is even more apparent in panel B, where even small features appear in identical manner on both figures. Moreover, there is no correspondence between the spectrum marked by the red dot in the 'Org' map in B and the map: from the signal intensity in the map, I'd expect a smaller peak in the corresponding spectrum.
The authors do indeed acknowledge a correlation between Org and Ca maps at lines 162-164, but the correspondence seems too remarkable to me, and the ambiguous correspondence between map and spectrum explained above makes me particularly doubtful. By contrast, there seems to be a greater correspondence map-spectra for Ca in Fig2B (assuming the 4 dots are placed in the same locations, since they are not marked for Ca).
A similar issue with maps vs. spectra for both Org and Ca also appears in Figure 3-C. Map for Org seems really noisy considering how sharp the edge used is, and map for Ca looks too black for the yellow and red points (I'd expect at least some noise considering the small peaks).

I also have general concerns regarding the quality of EELS data and the accuracy of their interpretation, as well as how information is presented:
1. Energy calibration: All peaks seem a bit shifted compared to expected values from well-reputed resources (https://eels.info/atlas), so I would like to see the alignment of the zero-loss peak (ZLP) to better judge. I'm not personally convinced about using specific peaks to do the energy calibration, and this should really be done based on the ZLP to avoid any pre-bias in peak interpretation/assignment. It's also not clear which peaks were used since a list of them is mentioned at lines 400-402, while in SI only the carbonate peak is mentioned (lines 11-12 SI).
2. Core-loss EELS vs. ELNES: this could be an interesting part of the paper but it's placed in SI and the 'carbonate peak' appears in the main manuscript without clear explanations. Overall, I find the presentation and treatment of absorption edges in the core-loss spectrum vs. ELNES peaks (after the C edge, for example the carbonate signature or the 287.5 eV peak used for map-plotting) treated in a confusing manner. Or at least I am presuming that the authors were attempting to do some ELNES, as this is not even clearly mentioned.
3. Chosen edges: given the centrality of elemental analyses in the different organelles, I wish the authors also examined other absorption edges especially for P (ie, the K-edge) since the L edge falls within the plasmon tail, which makes it hard to tell if it's not there or just hidden in the background (as the authors also seem acknowledging at lines 209-210). And why not look also at N in addition to C for a better representation of the organics?
4. Overall, I disagree with the terminology 'Org' for the EELS maps of the C-edge. They should simply be labelled as 'C', similarly to Ca and P, if that element edge is used. I'm not really sure what the chosen C peak really is (287.5 eV) – this should be better discussed, especially in relation to main C edge (see comment 2 for EELS vs ELNES). I'm aware that the authors discuss this in SI, but this is such a key part that it should be also reported in the main manuscript and described in a more convincing manner.

In general, data and images could be better presented. For example:
1. Figure 1: given the importance of different organelles and their locations especially for the model proposed, it would be nice to have consistency in low magnification overview images vs. high magnification insets. It is not clear to me how all these organelles relate to each other, except for 1-I. Some panels contain marked regions that are not shown in the rest of the figure, or at least not labelled like the rectangles in A and J. It is also a bit confusing to include a HAADF-STEM image where the rest of the panel is all BF-TEM.
2. Since the authors want to compare peak intensities, spectra from individual points should be plotted on the same y-axis and not staggered (Figures 2-4).
3. Figure S1-C is missing scale bars!
4. Why are EELS maps for P not shown in Figures 2 and 3?

Other aspects that leave me a bit puzzled are:
1. The authors seem quite excited about using a monochromator for getting high energy resolution data, but I'd like to point out that 150 meV is pretty standard for core-loss EELS – not that I think high spectral resolution matters so much here (and a NION should do better). There also seems to be a bit of confusion regarding spatial vs. energy resolution based on how lines 138-140 are worded.
2. Lines 116-117: "Several spectrum images were acquired from each type of organelles". This is not reflected by the content of neither the main manuscript or SI. It's a pity to collect lots of data and then just show spectra from a few points.
3. Line 197: "Identical EELS results were obtained from stained and unstained sections". This is not so well described in the manuscript, and overall it's also unclear whether the samples used for TEM, EDX, or EELS were the same given also the different thickness mentioned in the methodology section.
4. EDX appears a bit randomly and just for one type of organelle.
5. Overall, the mechanism proposed (represented in Figure 5) seems a bit ambitious based on the experimental evidence.

Version 1:

Reviewer comments:

Reviewer #1

(Remarks to the Author)
My specific comments have been addressed in a satisfactory manner. The scenario proposed in the manuscript is conceivable, and the authors are cautious in their conclusions. It would be interesting, however, to develop the discussion with an examination of what are possible alternative or additional pathways, besides those hypothesized. The issues raised by reviewer #2 are mainly technical issues about EELS, and are not within my expertise.

Reviewer #2

(Remarks to the Author)
Please refer to the attachment as I think it's easier to follow the replies to the authors' answers that way. I'm also copying the below just in case.

Fig2:
Mistakes can happen also when in good faith, and I feel it is my role as reviewer to make sure that the paper is of the highest quality possible, which is mostly to the authors' benefit. Unfortunately, this answer still does not solve my doubt, which concerns how the spectral images (maps) themselves were obtained. This is still not shown in this answer, where again the two maps look remarkably similar and the only difference is the level of background when extracting the line profile (which makes sense – it is noticeable even without doing this that the organic map has a higher background than the calcium map, but the trend in intensity variation is the same). I would like the authors to show the raw spectrum and the background and signal extraction windows for both edges of interest to make sure the background is being extracted properly for each, and that we don't see a 'ghost' signal from the organics in the calcium map (eg, from lack of background extraction prior to Ca edges). Again, this is not a matter of good faith or not, but of making sure that these rather complex data are properly processed. The authors should also include such representation of how raw data was treated in SI.

Spectra vs maps ('dots' Org vs Ca):
Perhaps my comment was not well worded. I understand that the maps are extracted from the same spectral image, but I was referring to the spectra shown as examples. Maybe this is a picky comment, but one can only assume that the 4 spectra chosen for Ca are in the same locations as those chosen for Org. In other words, even if the maps are extracted from the same spectral image, one could choose to show examples of individual spectra coming from different pixels/sub-regions in the image. Hence, you should make it clear where the 4 spectra for Ca come from. There seems to be a bit of confusion between spectral image vs. spectrum.

Peak sharpness:
I apologize for using a confusing terminology in my comment, as I was thinking about intensity (how much the peak 'stands out'), but I incorrectly referred to it as sharpness. The authors are right regarding sharpness, but then if we think about intensity I'm a bit unsure of the correlation between map and spectrum. This goes back to my first comment of making sure that background and edge intensity are extracted well.

Energy calibration:
Firstly, I would like to point out that this is a double-blinded revision so referring to your previous work or groups involved does not mean much - and I'd keep my comment as unbiased as possible regardless. My doubt partially remains: which peak was used to calibrate? One must have selected one specific peak (the carbonate?) and adjusted its position to calibrate all the remaining peaks. At lines 417-419 I still see a list of peaks mentioned specifically in reference to energy calibration. I am not asking about which peaks were considered for the study in general, but specifically how the calibration was done. I also do not understand why the authors could not use the ZLP with the same acquisition settings and then simply shift the energy range – I understand this cannot be done at once, but, to my knowledge, it could still be done in two steps. And in fact you do shift the energy range eg for Ca and C vs P, as you explain a few answers below. Please also be careful with correct terminology overall both in the manuscript and in these replies: here you're referring to energy dispersion of 100 eV, while I imagine you are actually referring to the energy range, as dispersion is measured in eV/channel and you report 0.1 eV/channel in the manuscript.

Maps for P and N:
The considerations on P and N reported here could perhaps be mentioned also in the manuscript, eg when you describe the chosen peaks.

Monochromator and energy resolution:
I am not saying that the monochromator is not useful/needed, nor that a higher energy resolution should be used – I totally agree that this is not feasible here. Hence my point: 150 meV is quite standard for core-loss EELS in monochromated instruments, so it should not really be a 'selling point' for this study, as it seems to be in some part of the paper. But to re-iterate, I don't think very high energy resolution is what matters here, so this is really not an essential discussion point. I think now it works better also considering the re-writing in the comment below.

Sample prep, staining:
I realize I may sound picky, but the text overall could be a bit smoother not to leave too much to the readers to interpret or figure out. For example, when you describe the preparation of TEM samples for imaging, you clearly mention using an ultramicrotome, while one can only assume you used the same method for preparing the EELS samples? Also the EELS samples are technically also TEM samples, so I guess a better distinction would be samples for imaging vs samples for

EELS. Since you also run a comparison between stained vs unstained, this should be included somewhere, at least in SI. And so what samples are shown in the images and spectra, stained or not? I stand by my comment that these aspects are not really clear.

Version 2:

Reviewer comments:

Reviewer #2

(Remarks to the Author)
The authors have addressed my comments, and I appreciate the effort and transparency in providing additional clarification regarding the EELS analysis. However, I remain perplexed as to how this analysis appears to have been performed. While the treatment of the Ca edge seems reasonable, the demonstration of the analysis of the ‘Org’ signal reinforces my concerns about the reliability of the reported results.
I do not find it appropriate to set the background extraction window within such a narrow portion of the spectrum. It is usually recommended that this window spans at least 10 channels (e.g., see the book by Williams and Carter), but here it appears to barely cover a single channel. Moreover, the background extraction should be performed before the onset of the main edge (C-K in this case), and not before individual near-edge features, as it was done here.
Therefore, given the background and signal extraction procedure shown in the rebuttal letter, it is unclear whether the resulting signal represents a genuine contribution of the sample composition. It seems possible that the extracted intensity reflects variations in the underlying spectrum (for example, mass-thickness contrast) rather than a true chemical signal. This could also explain the strong spatial overlap observed between the Org and Ca maps.
At this stage, I cannot provide any further constructive comments on this work. The demonstration of the analysis provided in the rebuttal letter reinforces my doubts regarding the robustness of the EELS analysis, which is an aspect too central to this work to be overlooked. I therefore do not consider the manuscript suitable for publication. If the editor nevertheless decides to proceed with publication against my recommendation and without involving an additional reviewer, I strongly recommend that the demonstrations of the background and signal extraction currently provided in the rebuttal letter for both Ca and C/Org be included in the supplementary information for full transparency to the readers.

Reviewer #3

(Remarks to the Author)
I have reviewed the manuscript, along with the comments of the authors and Reviewer 2 concerning the EELS analysis. Overall, while the reviewer has raised some excellent points and is right to demand transparency in the data analysis, I find the authors’ EELS analysis workflow to be robust, justified, and well within the accepted standards of the EELS community.

My impression is that the main source of contention surrounds the background subtraction on the organics peak, which due to its proximity to other fine structure in the carbon K edge, does not have a standard background fit. For me, it is very common to leverage non-standard background fits in such cases when peaks of interest are grouped so closely together. A linear fit from two narrow windows on either side of the peak is exactly what I would’ve done, and because it’s a linear fit you really don’t need the larger window that the reviewer suggests is necessary. Most importantly for me though is that the authors only draw qualitative conclusions that are easily visible from a cursory inspection of the un-background-subtracted spectra presented in Figure 2a. The maps are only used as visualization tools to demonstrate to the where the org peak is strong and where it’s not, so a robust background subtraction isn’t even necessary.

There are also some discussions surrounding calibration, but for me this is a non-issue. Calibrating from the ZLP and the dispersion and taking two known features and calibrating from those are both field-accepted calibration standards. One isn’t better than the other. Examining the author’s experimental setup, the first isn’t an option for them, so the strategy the authors have used is the only reliable calibration method for their measurements.

I believe this paper could be published as is, but I do have some final thoughts on what how the authors can avoid readers coming to the same conclusion as Reviewer 2.

1. I believe the authors could have conclusively addressed Reviewer 2’s points simply by including the map of the 285 eV onset of the C-K edge (as shown in Fig. S2) in the main text. I believe the reviewer was concerned that there was some background/thickness artifact that caused the 287.5 eV peak to follow the same intensity pattern as the Ca peak. Since the 285 eV peak will be subject to the same background/thickness effects as the 287.5 eV peak, the contrast reversal between these maps proves definitively that the background subtraction is finding real changes in bonding.

2. This paper would benefit greatly from using a high-contrast color scheme for the signal maps as opposed to grayscale. Also if they were to use different color maps for Org/Ca/P, respectively I think it will help readers see the maps as similar but not exactly similar. Most importantly, the maps need color bars so we know what 0 background really looks like (I strongly recommend the authors do this even if the remainder of my suggestions are ignored).

3. I also believe this paper would benefit from including some version of the background subtraction shown in the rebuttal document in the main text. Figure 2 is pretty sparse; you could zoom in on the 280-290 meV range for the Org peak (really nothing interesting happens between 290 and 310) and actually show dotted lines to indicate the background fit on all

sixteen spectra presented in Fig. 2. This would help non-EELS-experts visualize what they are supposed to be looking at.

Open Access This Peer Review File is licensed under a Creative Commons Attribution 4.0 International License, which permits use, sharing, adaptation, distribution and reproduction in any medium or format, as long as you give appropriate credit to the original author(s) and the source, provide a link to the Creative Commons license, and indicate if changes were made.
In cases where reviewers are anonymous, credit should be given to 'Anonymous Referee' and the source.
The images or other third party material in this Peer Review File are included in the article's Creative Commons license, unless indicated otherwise in a credit line to the material. If material is not included in the article's Creative Commons license and your intended use is not permitted by statutory regulation or exceeds the permitted use, you will need to obtain permission directly from the copyright holder.
To view a copy of this license, visit https://creativecommons.org/licenses/by/4.0/

Reviewer #1 (Remarks to the Author):

This is a very interesting manuscript, with beautifully performed experiments followed by thoughtful interpretation.

The authors applied advanced electron microscope techniques to examine the structure and composition of vesicles in cells directly involved in building the shell of a mollusk bivalve. To achieve their purpose, they used a combination of advanced electron microscopy techniques on samples prepared such that they cause minimal change, allowing the observation of samples in close to native conditions. Three vesicle types were analyzed, including bilayered vesicles, ribosome associated vesicles and mitochondria. The mitochondria contained calcium phosphate granules, and this evidence "together with the increasing evidence for the involvement of phosphate in regulating calcium carbonate-based systems, leads us to propose the existence of conserved biomineralization pathways from invertebrates to vertebrates, rooted in an ancestral mechanism of intracellular calcium regulation" (citation from the manuscript). This is a most important statement, supported by the first evidence of the presence of Ca/P containing mitochondrial granules in the shell building cells of mollusks. For this reason, together with the high quality of the work, I recommend acceptance of the paper in COMMSBIO.

The authors propose a complex model involving high cytoplasmic calcium concentration triggering formation of Ca/P mitochondrial granules, followed by mitophagy, autophagolysosome formation, and exocytosis, together with other parallel alternative pathways. As pointed out by the authors, with due caution, "Further evidence on calcium transport from CaP granules to the shell, and its transformation into the final precursor phase, is needed to support this hypothesis". This is especially true in the light of other studies that recorded phosphorus involvement in vesicles in sea urchin embryos (ref 37 in the manuscript), side by side with calcium extraction and concentration from sea water endocytosis. When, how and if a shift from calcium phosphate to carbonate occurs is the real mystery.

We sincerely appreciate your comments and are pleased that you found the article interesting.

Specific comments follow:

Page 4:

- 1mm size animals were selected. Please clarify if these are adults, veligers or larvae. This is important, because in larvae, the shell is known to be composed of amorphous calcium carbonate, and the shell deposition mechanism may be different. If the experiments were performed on larvae or veligers, do you believe that the conclusions of your work are valid also for adults?

Answer:

This stage should be considered juvenile based on their very small size (ca. 1 mm) and a morphology that shares most characteristics with the adult form (Lago et al., 10.1038/s41598-025-11439-3), except for the lack of sexual maturity. A clarification has been added in line 373: "Six juvenile *M. galloprovincialis* (exhibiting adult morphology but still sexually immature), approximately 1 mm in width…".

- A statement is made, that "Mantle cells are virtually in contact with the shell, separated only by a narrow extracellular compartment, known as the extrapallial space", and this is indeed what is observed, but I wonder whether this space may readjust from farther away to close contact depending on whether, where and when the shell mineral is being deposited at the time of sample preparation.

Answer:

Whether the dimensions of the extrapallial space could vary depending on shell mineralization remains a possibility, though it is uncertain. Accurately determining its exact dimensions is challenging, as sample preparation can induce distortions. As noted in the manuscript (line 77), in the region of the adductor muscle, where tendon cells are anchored to the shell via collagen fibers (Castro-Claros et al., 10.1016/j.actbio.2020.09.053), the extrapallial space is expected to be relatively more stable.

Page 8:

"A correlation between the intensities of the organic and calcium peaks was observed across the 162 different positions in both bilayered vesicles and RAVs (carbon and calcium curves in Fig. 2A and 2B)". I agree that there is a clear correlation between the curves in figure 2A, but I cannot see a good correlation in figure 2B. Please reconsider and explain

Answer:

The organic fraction is obtained from the intensity of the 287.5 eV peak and not from the total C-K signal. If we consider the 287.5 eV peak in Figure 2B, the spectra shown in different colors indicate that higher organic peaks are consistently associated with higher calcium peaks, while lower organic peaks correspond to lower calcium peaks. Moreover, the maps clearly show that the spatial distribution of both signals overlaps. For clarity, the explanation was added in the main text line 118-122 and in the caption of figure 2 (line 190-191). Additionally, labels indicating the 287.5 eV peak have been added to figures 2 and 3.

Reviewer #2 (Remarks to the Author):

The paper addresses the role of different organelles in mollusc shells mineralization, using STEM and EELS to demonstrate the presence of minerals in the organelles and propose a biomineralization pathway. While biomineralization pathways are of clear importance across a variety of organisms and the methodology used is interesting, I find certain aspects to be of great concern in the current form of the manuscript. In my opinion, these concerns are too fundamental to be addressed through revision, and therefore I cannot recommend this manuscript for publication.

My biggest concern regards Figure 2: it seems quite apparent to me that the EELS maps for the organics and calcium are indeed the same map with different brightness and contrast. This is even more apparent in panel B, where even small features appear in identical manner on both figures. Moreover, there is no correspondence between the spectrum marked by the red dot in the 'Org' map in B and the map: from the signal intensity in the map, I'd expect a smaller peak in the corresponding spectrum.

The authors do indeed acknowledge a correlation between Org and Ca maps at lines 162-164, but the correspondence seems too remarkable to me, and the ambiguous correspondence between map and spectrum explained above makes me particularly doubtful.

Answer:

We were very surprised by these comments. To demonstrate our good faith, we provide below the histograms of each image, as well as cross-sections of the organic and calcium maps at the same positions. They clearly show that the images are genuine and not the same one displayed with different brightness or contrast. Furthermore, the raw data can be provided for verification.

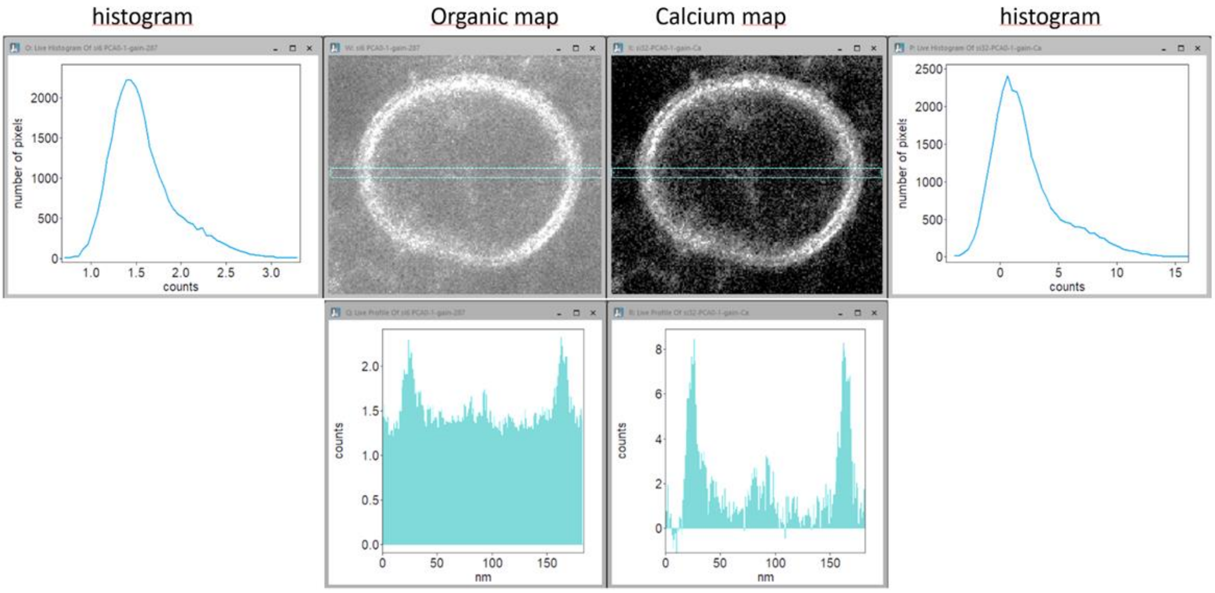

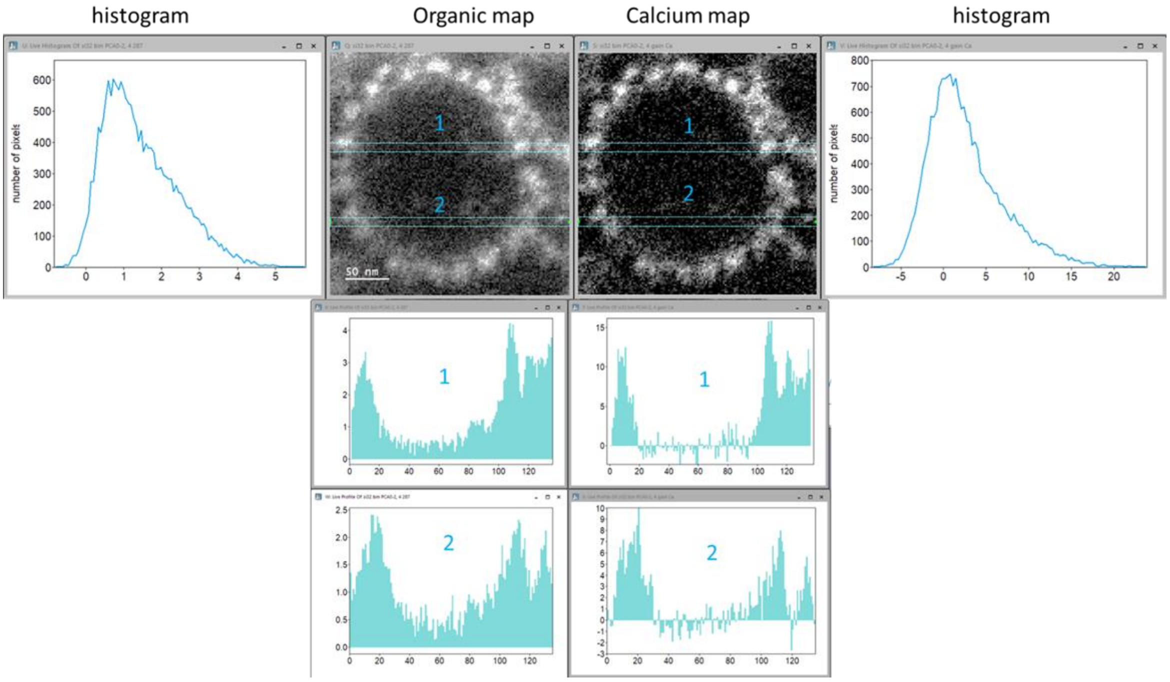

By contrast, there seems to be a greater correspondence map-spectra for Ca in Fig2B (assuming the 4 dots are placed in the same locations, since they are not marked for Ca).

Answer:

The comment "assuming the 4 dots are placed in the same locations, since they are not marked for Ca" is not justified: there is no need to place dots in calcium maps because the carbon and calcium signals are obtained at each position simultaneously from a single spectrum because we use an energy dispersion of 100 eV that covers both edges. This was specified in the Materials and Methods section at lines 413-415.

A similar issue with maps vs. spectra for both Org and Ca also appears in Figure 3-C. Map for Org seems really noisy considering how sharp the edge used is, and map for Ca looks too black for the yellow and red points (I'd expect at least some noise considering the small peaks).

Answer:

The sharpness of a peak observed at a single point does not define the spatial distribution in the corresponding maps. The contrast arises from differences in signal intensity between adjacent areas. The organic signal within the granules is not significantly higher than the background signal extracted from the surroundings. Similarly, since the calcium and phosphorus contents are markedly higher inside the granules and their surroundings contain much lower amounts of these elements (some bright areas can still be observed when zooming in), the background appears darker.

I also have general concerns regarding the quality of EELS data and the accuracy of their interpretation, as well as how information is presented:

1. Energy calibration: All peaks seem a bit shifted compared to expected values from well-reputed resources (https://eels.info/atlas), so I would like to see the alignment of the zero-loss peak (ZLP) to better judge. I'm not personally convinced about using specific peaks to do the energy calibration, and this should really be done based on the ZLP to avoid any pre-bias in peak interpretation/assignment.

Answer:

The use of specific peaks to calibrate the EELS spectrum is the most commonly used method in the literature because it provides the highest precision for peak

identification. For instance, it is used in biomineral studies by Nitiputri et al. (10.1021/acsnano.6b02443),Kłosowski et al. (10.1021/acsbiomaterials.6b00300) and in all our previous publications, some of which have been published in high-impact journals (de Frutos et al., 10.1021/acsnano.2c11169; Gay et al., 10.1021/acsnano.9b07664; Ajili et al., 10.1038/s41467-022-29169-9; Ajili et al., 10.1021/acs.chemmater.3c01169)

We would like to point out that the team of Q. Ramasse (https://eps.leeds.ac.uk/dir-record/research-projects/4265/core-equipment superstem-2020) and D. McComb (https://profiles.imperial.ac.uk/d.mccomb) are among the world leaders in EELS approach.

There are several important reasons for that:

1) The EELS Atlas provided by Gatan/Ametek company (https://eels.info/atlas) is a reference commonly used in EELS laboratories for an initial rough estimation of elemental edge positions. It is a collaborative resource that does not provide the sources of the data, so it is not possible to determine how the data have been obtained and calibrated. Moreover, EELS atlas does not provide the precise edge positions for a given compound, as these depend on the chemical bonding environment of the element, and not all possible compounds are included in the atlas. For instance, no information is provided for calcium phosphate compounds and data given for calcium are from calcium carbonate with no mention of the phase (calcite or aragonite). A limited number of EELS studies can be found in the literature concerning biominerals and biological systems. In contrast, a large amount of XANES data has been published for these materials, providing reliable information for the different compounds and phases. Edge positions in XANES are exactly the same as EELS ones. This is the reason why this method is usually chosen for this type of study.

2) If ZLP is used for calibration, the ZLP must be recorded in the same spectrum than the edges of interest (carbon and calcium edges at 285 eV and 350eV, respectively). Then, the dispersion has to be chosen to cover the energy range from ZLP to the elemental edges. However, the spectral resolution of an EELS spectrum is not determined by the electron source resolution but also by the dispersion chosen for the acquisition. Our EELS timepix direct detector has 1000 pixels. This requires a dispersion of ≈400 eV which limits the energy resolution to 0.4 eV/px which is not compatible with high spectral resolution

analysis of the spectral signatures. In the present study, the dispersion was set to 100 eV to record signal around carbon and calcium edges (285 eV and 350 eV) in order to achieve the best spectral resolution possible.

An additional reason to use specific peaks for a precise calibration in the energy range of the edges of interest is related to the non-linearity of the energy dispersion of EELS spectrometers.

It's also not clear which peaks were used since a list of them is mentioned at lines 400-402, while in SI only the carbonate peak is mentioned (lines 11-12 SI).

Answer:

This suggestion has been taken into account. The peaks used for the study are mentioned on the main text at lines 113-120. We had a sentence to specify the position of carbonate peak in the main text at line 118. For greater clarity, we also added an additional mention to the organic peak at 287.5 eV at line 119 and in Figure 2 caption (line 190-191).

2. Core-loss EELS vs. ELNES: this could be an interesting part of the paper but it's placed in SI and the 'carbonate peak' appears in the main manuscript without clear explanations. Overall, I find the presentation and treatment of absorption edges in the core-loss spectrum vs. ELNES peaks (after the C edge, for example the carbonate signature or the 287.5 eV peak used for map-plotting) treated in a confusing manner. Or at least I am presuming that the authors were attempting to do some ELNES, as this is not even clearly mentioned.

Answer:

Part of the explanations given in SI have been transferred to the main text at lines 114-122: "The carbon spectra exhibited a series of sharp peaks associated with the C 1s $\rightarrow \pi^*$ transitions, along with a broad shoulder around 300 eV, characteristic for C 1s $\rightarrow \sigma^*$ transitions. The positions of the sharp peaks vary depending on the carbon bonding environment. This near-edge fine structure can be used to analyze the different compounds. The carbonate is detected on the carbon edge by the presence of a peak at 290.3 eV. As in previous EELS studies concerning biominerals, the distribution of organic compounds was extracted from the peak at 287.5 eV on the C-K edge (cf. SI Appendix, Fig. S1). The phosphorus and calcium maps were obtained from the integration of the corresponding elemental edges after background subtraction, while the different

carbon compounds were mapped based on the intensities of their characteristic peaks.”.

3. Chosen edges: given the centrality of elemental analyses in the different organelles, I wish the authors also examined other absorption edges especially for P (ie, the K-edge) since the L edge falls within the plasmon tail, which makes it hard to tell if it’s not there or just hidden in the background (as the authors also seem acknowledging at lines 209-210). And why not look also at N in addition to C for a better representation of the organics?

Answer:

To the best of our knowledge, the K-edge of phosphorus has not been used previously in the literature for EELS analysis due to its very high energy position (>2100 eV), its signal will be much lower than the P-L_{23} edge and nearly impossible to detect because EELS spectrometers are not designed for these high energies. For instance, the K-edge spectrum is not given in the EELS Atlas (https://eels.info/atlas).

Concerning N, we demonstrated in a previous study that the maps were identical to the ones obtained from the 287.5 eV carbon peak but the signal from N was much noisier than the carbon one due to the low intensity of N signal (Gay et al., 10.1021/acsnano.9b07664).

Another reason is that we tried to minimize the electron dose applied to each area to limit the damage as much as possible. Here, we recorded two spectra on each area: a first one covering the carbon + calcium range (energy range of 100 eV) and a second one for the phosphorus range (energy range of 100 eV). As nitrogen is more than 100 eV distant from carbon, we should repeat a third measurement at the same specimen position, inducing more damage. Alternatively, the energy range should be increased to 200 eV resulting in a reduction of the energy resolution. Therefore, the optimal approach was to analyze the organic compounds using the carbon edge.

4. Overall, I disagree with the terminology ‘Org’ for the EELS maps of the C-edge. They should simply be labelled as ‘C’, similarly to Ca and P, if that element edge is used. I’m not really sure what the chosen C peak really is (287.5 eV) – this should be better discussed, especially in relation to main C edge (see

comment 2 for EELS vs ELNES). I'm aware that the authors discuss this in SI, but this is such a key part that it should be also reported in the main manuscript and described in a more convincing manner.

Answer:

There seems to be a misunderstanding here. "Org" is not used to label the entire C-edge signal, but only the small peak at 287.5 eV, which was previously assigned to organic compounds. Please also refer to the answer to question 2 and the corresponding modifications made in the text.

In general, data and images could be better presented. For example:

1. Figure 1: given the importance of different organelles and their locations especially for the model proposed, it would be nice to have consistency in low magnification overview images vs. high magnification insets. It is not clear to me how all these organelles relate to each other, except for 1-I.

Answer:

The low-magnification images in Figure 1 (A-C, J), show the different organelles correctly labelled and clearly localized next to the shell. Low magnification overview images vs. high magnification insets are provided for Figs. 1A-B; 1E-F; 1H-I; 1J-K.

The connection between the mitochondrion and the RAV (Fig. 1-I) is the only direct interaction between vesicles shown in this study and the only one considered in the proposed model.

Some panels contain marked regions that are not shown in the rest of the figure, or at least not labelled like the rectangles in A and J. It is also a bit confusing to include a HAADF-STEM image where the rest of the panel is all BF-TEM.

Answer:

We have taken it into account and we have modified the labels in Figures 1A and 1J to maintain consistency.

The inclusion of an HAADF-STEM image is intended to clearly show the bilayer membranes and the vesicular content of the multivesicular body. Otherwise, in TEM images, the contrast may not be sufficient to clearly reveal these structures.

2. Since the authors want to compare peak intensities, spectra from individual points should be plotted on the same y-axis and not staggered (Figures 2-4).

Answer:

This representation is commonly used in all EELS studies (Nitiputri et al., 10.1021/acsnano.6b02443; Klosowski et al., 10.1038/s41598-018-20072-2; Klosowski et al., 10.1021/acsbiomaterials.6b00300; Ajili et al., 10.1038/s41467-022-29169-9; de Frutos et al., 10.1021/acsnano.2c11169) because it provides a better view of the different spectra.

3. Figure S1-C is missing scale bars!

Answer:

A scale bar (valid for all panels) was added.

4. Why are EELS maps for P not shown in Figures 2 and 3?

Answer:

Maps are not provided for bilayered vesicles and RAV because the signal is much weaker than that of mitochondrial granules and would be lost in the noise. In Figure 4, to obtain a measurable signal, it was summed over a (6×6 px^2) area.

Other aspects that leave me a bit puzzled are:
1. The authors seem quite excited about using a monochromator for getting high energy resolution data, but I'd like to point out that 150 meV is pretty standard for core-loss EELS – not that I think high spectral resolution matters so much here (and a NION should do better).

Answer:

We strongly disagree with this statement. A resolution of 150 meV in core loss EELS is definitely not accessible without a monochromator. The best electron sources are field emission guns, which provide electrons with an energy dispersion of approximately 300 meV. This value is further degraded to about 600 meV by the point-spread-function of the EELS detector when a CCD is used, as is common in most microscopes. This is for instance the case of the Titan 80–300

microscope at the Imperial College (Kłosowski et al.,10.1021/acsbiomaterials.6b00300). Our monochromated Nion Hermes with the use of a Timepix direct detector provides an energy resolution down to ≈10 meV but only in the EELS low loss range (Chaupard et al, 10.1021/acsnano.2c09571). For this, the dispersion has to be adjusted below 10 eV (0.01eV/px) and this is not compatible with core loss measurements. As a reminder, in this study we used an energy range of 100 eV for the measurements in order to analyse carbon and calcium edges simultaneously.

There also seems to be a bit of confusion regarding spatial vs. energy resolution based on how lines 138-140 are worded.

Answer:

Thanks to the reviewer for the correction of this mistake. The sentence has been rewritten (line 145-148): "the use of monochromated STEM-EELS provides improved spectral resolution and a higher signal-to-noise ratio thanks to the Timepix3 direct electron detector. Combined with the high spatial resolution of this approach, it enables mapping of different compound distributions at nanometric resolution."

2. Lines 116-117: "Several spectrum images were acquired from each type of organelles". This is not reflected by the content of neither the main manuscript or SI. It's a pity to collect lots of data and then just show spectra from a few points.

Answer:

Additional spectral images for each organelle have been included in the SI (Fig. S2).

3. Line 197: "Identical EELS results were obtained from stained and unstained sections". This is not so well described in the manuscript, and overall it's also unclear whether the samples used for TEM, EDX, or EELS were the same given also the different thickness mentioned in the methodology section.

Answer:

We are confused about this comment. It is clearly stated in the Methods section that we used different section thicknesses for EDX and TEM (100 nm) and for EELS (50 nm). The reason for this is that EELS is not compatible with 100 nm

sections, as the signal drops to nearly zero at that thickness due to multiple scattering.

Regarding staining, some sections were stained while others were not. In the unstained sections, only the electron-dense granules were visible and there was no difference in spectra compared to stained sections. Bilayered vesicles and RAV were not visible on unstained sections, so we were obliged to analyse them on stained sections. The good agreement obtained on mitochondrial granules with and without staining demonstrates no influence of staining on EELS data. A sentence has been added at line 208: "EELS analyses of electron-dense granules were performed on stained and unstained sections. No differences were observed in the EELS spectra in both conditions.".

4. EDX appears a bit randomly and just for one type of organelle.

Answer:

We used EDX mainly to obtain the Ca/P ratio in the electron-dense granules (lines 398-400) because for the other structures the signal was too low to be detected. Because of their high Ca and P content, the EDX maps displayed good resolution, so we found it useful to include them.

5. Overall, the mechanism proposed (represented in Figure 5) seems a bit ambitious based on the experimental evidence.

Answer:

The model, as indicated in line 337 of the main text, is grounded in the results of this study and is consistent with the numerous bone studies cited throughout the manuscript. We consider it highly useful, as it provides a clear schematic representation of our proposal. A clarification has also been included in the caption of Figure 5 (lines 353-354).

Reviewers' comments:

Reviewer #1 (Remarks to the Author):

My specific comments have been addressed in a satisfactory manner. The scenario proposed in the manuscript is conceivable, and the authors are cautious in their conclusions. It would be interesting, however, to develop the discussion with an examination of what are possible alternative or additional pathways, besides those hypothesized. The issues raised by reviewer #2 are mainly technical issues about EELS, and are not within my expertise.

We appreciate this positive comment. Alternative or additional pathways cannot be ruled out, as acknowledged in the text, but we believe that in order to achieve a clear and coherent discussion, it is preferable to frame our observations within the context of the existing literature. Expanding the discussion with additional alternatives could be of interest in a future review.

Reviewer #2 (Remarks to the Author):

Please refer to the attachment as I think it's easier to follow the replies to the authors' answers that way. I'm also copying the below just in case.

Fig2:
Mistakes can happen also when in good faith, and I feel it is my role as reviewer to make sure that the paper is of the highest quality possible, which is mostly to the authors' benefit. Unfortunately, this answer still does not solve my doubt, which concerns how the spectral images (maps) themselves were obtained. This is still not shown in this answer, where again the two maps look remarkably similar and the only difference is the level of background when extracting the line profile (which makes sense – it is noticeable even without doing this that the organic map has a higher background than the calcium map, but the trend in intensity variation is the same). I would like the authors to show the raw spectrum and the background and signal extraction windows for both edges of interest to make sure the background is being extracted properly for each, and that we don't see a 'ghost' signal from the organics in the calcium map (eg, from lack of background extraction prior to Ca edges). Again, this is not a matter of good faith or not, but of making sure that these rather complex data are properly processed. The authors should also include such representation of how raw data was treated in SI.

The raw data (without any processing) are accessible to the reviewer at the following link:
https://zenodo.org/records/18668299?token=eyJhbGciOiJIUzUxMiJ9.eyJpZCI6ImI3ZDhl

YTE4LWVkZmQtNDE0OC1iYWZiLTU3NTllYTQwNTM1YyIsImRhdGEiOnt9LCJyYW5kb20iOiIzOGE1NjhmZDMyMmZjODNjZGZlZWVjZjI2YWMzZTU2NCJ9.ah1wcjkKDPKiJVRKk3rp8tL8BHTZEwvdGRkSRAgRcqlDBqodTsoJmwGW0mGIEP8EwRunnRkuBZaexGgEvxuOfg . As we indicated in the Material and Methods section, raw data were too noisy to be used directly for chemical mapping and they were binned, denoised using principal component analysis (PCA), and gain corrected. We encourage the reviewer to do the same if he wishes to check the validity of our processing protocol.

Maps were extracted after background subtraction. We provide below the figures explaining how the background is subtracted: for calcium the background is fitted by a power law before and after the Ca edge; for organic compounds, the intensity of the peak at 287 eV is obtained by subtraction of a linear law to remove the other contributions to carbon edge. For both spectral features, the green-shaded area was integrated between the two red boxes.

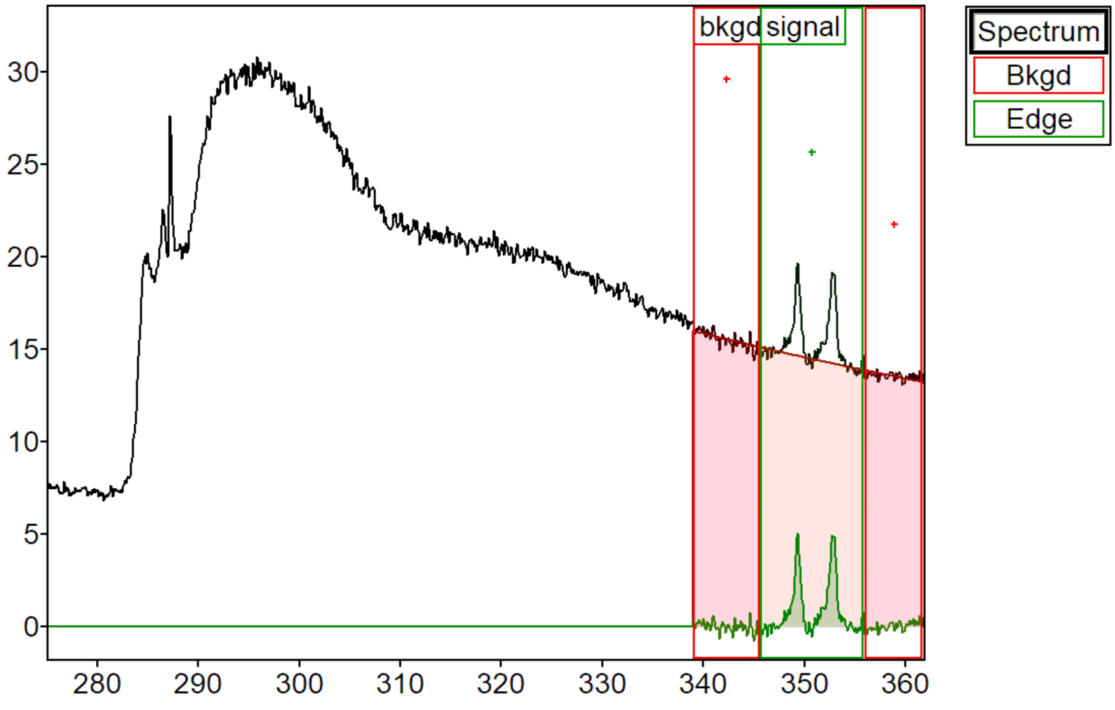

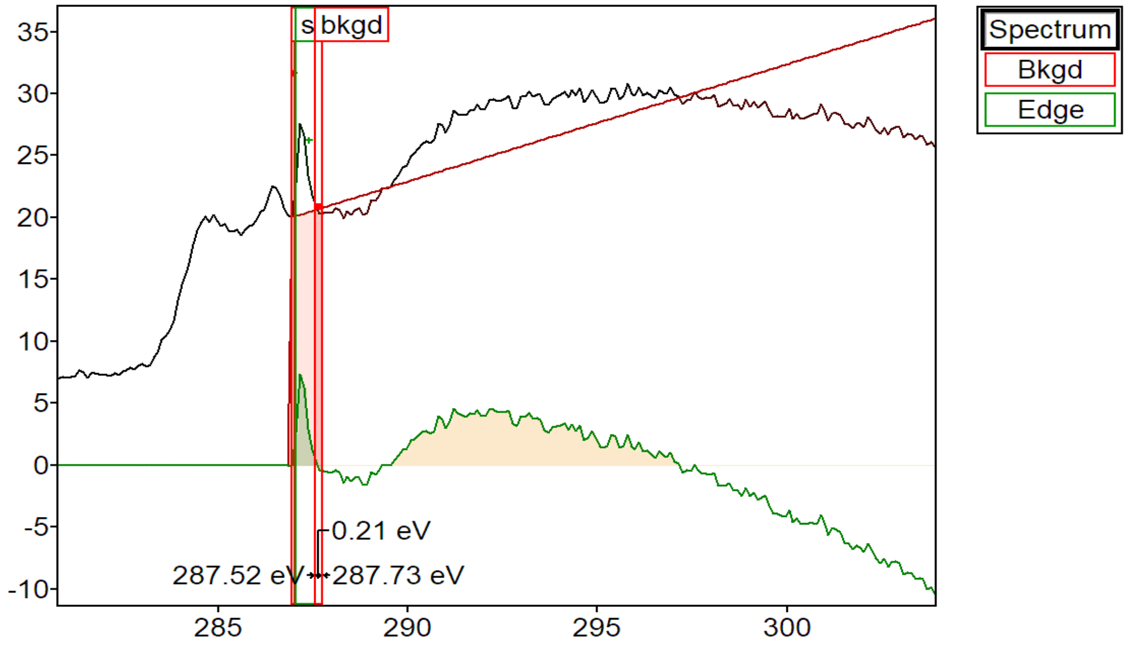

Spectra vs maps ('dots' Org vs Ca):
Perhaps my comment was not well worded. I understand that the maps are extracted from the same spectral image, but I was referring to the spectra shown as examples. Maybe this is a picky comment, but one can only assume that the 4 spectra chosen for Ca are in the same locations as those chosen for Org. In other words, even if the maps are extracted from the same spectral image, one could choose to show examples of individual spectra coming from different pixels/sub-regions in the image. Hence, you should make it clear where the 4 spectra for Ca come from. There seems to be a bit of confusion between spectral image vs. spectrum.

We have positively considered the comment and have also added the location points to the Ca map.

Peak sharpness:
I apologize for using a confusing terminology in my comment, as I was thinking about intensity (how much the peak ‘stands out’), but I incorrectly referred to it as sharpness. The authors are right regarding sharpness, but then if we think about intensity I’m a bit unsure of the correlation between map and spectrum. This goes back to my first comment of making sure that background and edge intensity are extracted well.

We hope that our response to the previous comment has clarified this aspect. The 287eV signal comes from the integral of a very small peak. As we already indicated in our previous answer to the reviewer, the organic signal within the

granules is not significantly higher than the background signal extracted from the surroundings. This explains why the corresponding map is so noisy

Energy calibration:
Firstly, I would like to point out that this is a double-blinded revision so referring to your previous work or groups involved does not mean much - and I'd keep my comment as unbiased as possible regardless. My doubt partially remains: which peak was used to calibrate? One must have selected one specific peak (the carbonate?) and adjusted its position to calibrate all the remaining peaks.

At lines 417-419 I still see a list of peaks mentioned specifically in reference to energy calibration. I am not asking about which peaks were considered for the study in general, but specifically how the calibration was done. I also do not understand why the authors could not use the ZLP with the same acquisition settings and then simply shift the energy range – I understand this cannot be done at once, but, to my knowledge, it could still be done in two steps. And in fact you do shift the energy range eg for Ca and C vs P, as you explain a few answers below.

One peak is not enough to calibrate EELS spectra for two reasons: (i) the nominal value of the dispersion is not sufficiently precise for proper calibration (due to the accuracy of reading the voltages applied to the electronic devices) so we need two reference peaks to calculate it with a high enough precision (few tenth of eV). In our experiments, the nominal value was 0.1eV/pxl and the value obtained from a pure calcite reference was 0.105eV/pxl. This difference results in a shift of 5eV over the 100eV energy range; (ii) Another problem comes from non-linearity of the relation between the "real" energy of the edges and their positions on the spectrum detected after the spectrometer dispersion. As explained by Webster et al. Ultramicroscopy (2020) https://doi.org/10.1016/j.ultramic.2020.113069 (fourth paragraph of the introduction and the associated references), this non-linearity arises from spectrometer optical aberrations which are only partially corrected even in the latest generation instruments, such as the one we use. The non-linearity is dependent on experimental conditions and vary with experimental adjustments. As a consequence of this non-linearity, the same calibration (linear) does not apply across the entire spectral range, which is why standard EELS calibration procedures rely on the use of two reference peaks within the energy range of interest. For these two reasons, using ZLP (very far from the energy range of interest) to calibrate the spectra will result in a tremendous shift.

We modified the text line 418 and 419 to precise which peaks are used for each energy range: "the carbonate peak at 290.3 eV, the calcium L□-edge peak at 349.3 eV for the energy range 255–360 eV, and the phosphorus L□, □-edge peaks at 138.5 eV and 147 eV for the energy range 90–195 eV".

Please also be careful with correct terminology overall both in the manuscript and in these replies: here you're referring to energy dispersion of 100 eV, while I imagine you are actually referring to the energy range, as dispersion is measured in eV/channel and you report 0.1 eV/channel in the manuscript.

Thank you for this comment. We check the complete manuscript and found no mistake. We apologize for the errors made in our previous responses to the reviewer and have taken care in the present document to avoid repeating them.

Maps for P and N:
The considerations on P and N reported here could perhaps be mentioned also in the manuscript, eg when you describe the chosen peaks.

Thank you for this comment. A comment for N was added line 30 in the SI. Regarding the P K-edge, we found no EELS studies in the literature that rely on this edge; therefore, we consider it unnecessary to mention it in the manuscript.

Monochromator and energy resolution:
I am not saying that the monochromator is not useful/needed, nor that a higher energy resolution should be used – I totally agree that this is not feasible here. Hence my point: 150 meV is quite standard for core-loss EELS in monochromated instruments, so it should not really be a 'selling point' for this study, as it seems to be in some part of the paper. But to re-iterate, I don't think very high energy resolution is what matters here, so this is really not an essential discussion point. I think now it works better also considering the re-writing in the comment below.

To lower the insistence to high resolution, line 108, the mention "high spectral resolution EELS" has been replaced by "monochromated EELS". We re-wrote also lines 136 to 150 to try to explain the differences between our present study compared to our previous ones (monochromated vs non-monochromated EELS, use of timepix direct detector to be sensitive to low signals), without overselling the "high spectral resolution". Now the term "high spectral resolution" is only used once in the manuscript (line 111).

Sample prep, staining:
I realize I may sound picky, but the text overall could be a bit smoother not to leave too much to the readers to interpret or figure out. For example, when you describe the preparation of TEM samples for imaging, you clearly mention using an ultramicrotome, while one can only assume you used the same method for preparing the EELS samples? Also the EELS samples are technically also TEM

samples, so I guess a better distinction would be samples for imaging vs samples for EELS.

For clarity, we have specified the use of ultramicrotome in each section (lines 396 and 404).

Since you also run a comparison between stained vs unstained, this should be included somewhere, at least in SI. And so what samples are shown in the images and spectra, stained or not? I stand by my comment that these aspects are not really clear.

We only did it for electron-dense granules, which were clearly visible in unstained sections, as mentioned in line 85. Other studied organelles were not visible in unstained specimens, reason why we used only stained ones. We have added a comparison between spectra from stained and unstained sections in the SI.3

Response to Reviewer 3

Reviewer #3 (Remarks to the Author):

I have reviewed the manuscript, along with the comments of the authors and Reviewer 2 concerning the EELS analysis. Overall, while the reviewer has raised some excellent points and is right to demand transparency in the data analysis, I find the authors’ EELS analysis workflow to be robust, justified, and well within the accepted standards of the EELS community.

My impression is that the main source of contention surrounds the background subtraction on the organics peak, which due to its proximity to other fine structure in the carbon K edge, does not have a standard background fit. For me, it is very common to leverage non-standard background fits in such cases when peaks of interest are grouped so closely together. A linear fit from two narrow windows on either side of the peak is exactly what I would’ve done, and because it’s a linear fit you really don’t need the larger window that the reviewer suggests is necessary. Most importantly for me though is that the authors only draw qualitative conclusions that are easily visible from a cursory inspection of the un-background-subtracted spectra presented in Figure 2a. The maps are only used as visualization tools to demonstrate to the where the org peak is strong and where it’s not, so a robust background subtraction isn’t even necessary.

There are also some discussions surrounding calibration, but for me this is a non-issue. Calibrating from the ZLP and the dispersion and taking two known features and calibrating from those are both field-accepted calibration standards. One isn’t better than the other. Examining the author’s experimental setup, the first isn’t an option for them, so the strategy the authors have used is the only reliable calibration method for their measurements.

I believe this paper could be published as is, but I do have some final thoughts on what how the authors can avoid readers coming to the same conclusion as Reviewer 2.

1. I believe the authors could have conclusively addressed Reviewer 2’s points simply by including the map of the 285 eV onset of the C-K edge (as shown in Fig. S2) in the main text. I believe the reviewer was concerned that there was some background/thickness artifact that caused the 287.5 eV peak to follow the same intensity pattern as the Ca peak. Since the 285 eV peak will be subject to the same background/thickness effects as the 287.5 eV peak, the contrast reversal between these maps proves definitively that the background subtraction is finding real changes in bonding.

2. This paper would benefit greatly from using a high-contrast color scheme for the signal maps as opposed to grayscale. Also if they were to use different color maps for Org/Ca/P, respectively I think it will help readers see the maps as similar but not exactly similar. Most importantly, the maps need color bars so we know what 0 background really looks like (I strongly recommend the authors do this even if the remainder of my suggestions are ignored).

3. I also believe this paper would benefit from including some version of the background subtraction shown in the rebuttal document in the main text. Figure 2 is pretty sparse; you could zoom in on the 280-290 meV range for the Org peak (really nothing interesting happens between 290 and 310) and actually show dotted lines to indicate the background fit on all sixteen spectra presented in Fig. 2. This would help non-EELS-experts visualize what they are supposed to be looking at.

We sincerely thank you for your careful review and valuable suggestions.

We have implemented all the recommended changes in Figures 2 and 3, including the 285 eV maps, the lines indicating the background subtraction, and the intensity scales. In addition, we have included the color maps in the Supplementary Material.

We believe these modifications have improved the clarity and quality of the manuscript.

Reviewer #2 (Remarks to the Author):

The paper addresses the role of different organelles in mollusc shells mineralization, using STEM and EELS to demonstrate the presence of minerals in the organelles and propose a biomineralization pathway. While biomineralization pathways are of clear importance across a variety of organisms and the methodology used is interesting, I find certain aspects to be of great concern in the current form of the manuscript. In my opinion, these concerns are too fundamental to be addressed through revision, and therefore I cannot recommend this manuscript for publication.

My biggest concern regards Figure 2: it seems quite apparent to me that the EELS maps for the organics and calcium are indeed the same map with different brightness and contrast. This is even more apparent in panel B, where even small features appear in identical manner on both figures. Moreover, there is no correspondence between the spectrum marked by the red dot in the 'Org' map in B and the map: from the signal intensity in the map, I'd expect a smaller peak in the corresponding spectrum.

The authors do indeed acknowledge a correlation between Org and Ca maps at lines 162-164, but the correspondence seems too remarkable to me, and the ambiguous correspondence between map and spectrum explained above makes me particularly doubtful.

Answer:

We were very surprised by these comments. To demonstrate our good faith, we provide below the histograms of each image, as well as cross-sections of the organic and calcium maps at the same positions. They clearly show that the images are genuine and not the same one displayed with different brightness or contrast. Furthermore, the raw data can be provided for verification.

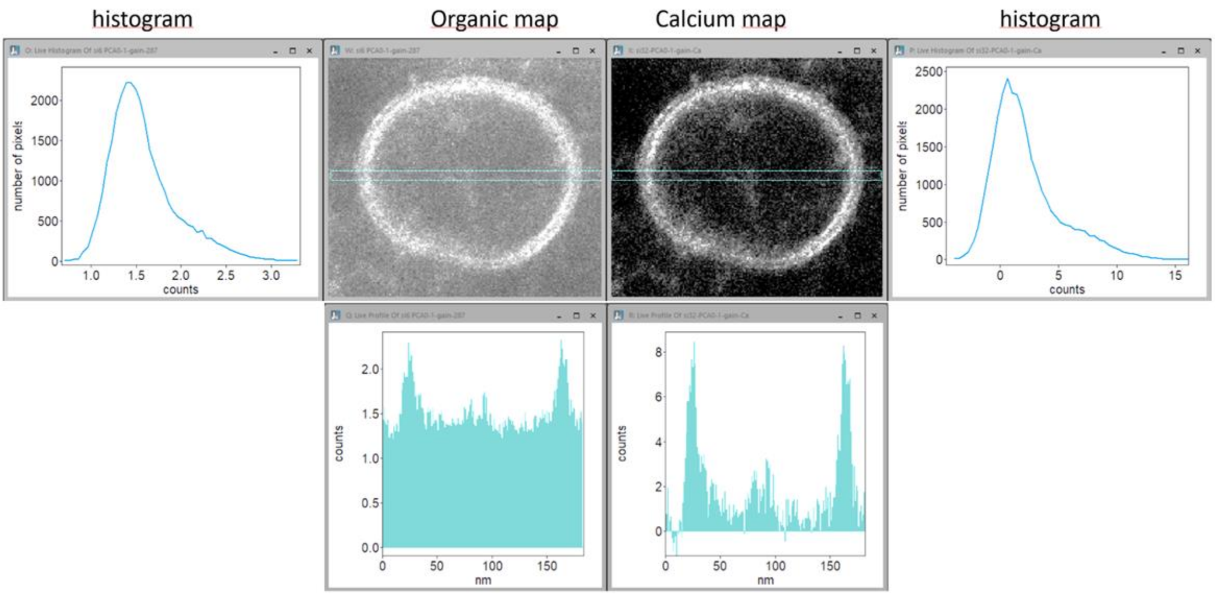

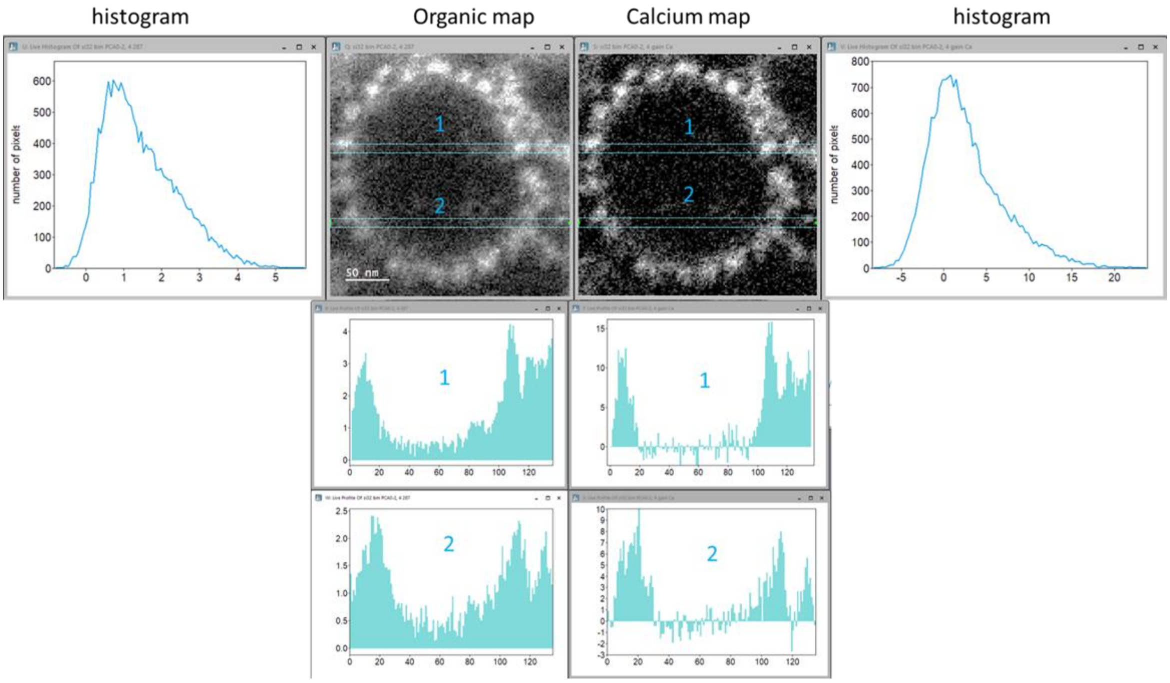

Mistakes can happen also when in good faith, and I feel it is my role as reviewer to make sure that the paper is of the highest quality possible, which is mostly to the authors' benefit. Unfortunately, this answer still does not solve my doubt, which concerns how the spectral images (maps) themselves were obtained. This is still not shown in this answer, where again the two maps look remarkably similar and the only difference is the level of background when extracting the line profile (which makes sense – it is noticeable even without doing this that the organic map has a higher background than the calcium map, but the trend in

intensity variation is the same). I would like the authors to show the raw spectrum and the background and signal extraction windows for both edges of interest to make sure the background is being extracted properly for each, and that we don't see a 'ghost' signal from the organics in the calcium map (eg, from lack of background extraction prior to Ca edges). Again, this is not a matter of good faith or not, but of making sure that these rather complex data are properly processed. The authors should also include such representation of how raw data was treated in SI.

By contrast, there seems to be a greater correspondence map-spectra for Ca in Fig2B (assuming the 4 dots are placed in the same locations, since they are not marked for Ca).

Answer:

The comment "assuming the 4 dots are placed in the same locations, since they are not marked for Ca" is not justified: there is no need to place dots in calcium maps because the carbon and calcium signals are obtained at each position simultaneously from a single spectrum because we use an energy dispersion of 100 eV that covers both edges. This was specified in the Materials and Methods section at lines 413-415.

Perhaps my comment was not well worded. I understand that the maps are extracted from the same spectral image, but I was referring to the spectra shown as examples. Maybe this is a picky comment, but one can only assume that the 4 spectra chosen for Ca are in the same locations as those chosen for Org. In other words, even if the maps are extracted from the same spectral image, one could choose to show examples of individual spectra coming from different pixels/sub-regions in the image. Hence, you should make it clear where the 4 spectra for Ca come from. There seems to be a bit of confusion between spectral image vs. spectrum.

A similar issue with maps vs. spectra for both Org and Ca also appears in Figure 3-C. Map for Org seems really noisy considering how sharp the edge used is, and map for Ca looks too black for the yellow and red points (I'd expect at least some noise considering the small peaks).

Answer:

The sharpness of a peak observed at a single point does not define the spatial distribution in the corresponding maps. The contrast arises from differences in

signal intensity between adjacent areas. The organic signal within the granules is not significantly higher than the background signal extracted from the surroundings. Similarly, since the calcium and phosphorus contents are markedly higher inside the granules and their surroundings contain much lower amounts of these elements (some bright areas can still be observed when zooming in), the background appears darker.

I apologize for using a confusing terminology in my comment, as I was thinking about intensity (how much the peak 'stands out'), but I incorrectly referred to it as sharpness. The authors are right regarding sharpness, but then if we think about intensity I'm a bit unsure of the correlation between map and spectrum. This goes back to my first comment of making sure that background and edge intensity are extracted well.

I also have general concerns regarding the quality of EELS data and the accuracy of their interpretation, as well as how information is presented:

1. Energy calibration: All peaks seem a bit shifted compared to expected values from well-reputed resources (https://eels.info/atlas), so I would like to see the alignment of the zero-loss peak (ZLP) to better judge. I'm not personally convinced about using specific peaks to do the energy calibration, and this should really be done based on the ZLP to avoid any pre-bias in peak interpretation/assignment.

Answer:

The use of specific peaks to calibrate the EELS spectrum is the most commonly used method in the literature because it provides the highest precision for peak identification. For instance, it is used in biomineral studies by Nitiputri et al. (10.1021/acsnano.6b02443),Kłosowski et al. (10.1021/acsbiomaterials.6b00300) and in all our previous publications, some of which have been published in high-impact journals (de Frutos et al., 10.1021/acsnano.2c11169; Gay et al., 10.1021/acsnano.9b07664; Ajili et al., 10.1038/s41467-022-29169-9; Ajili et al., 10.1021/acs.chemmater.3c01169)

We would like to point out that the team of Q. Ramasse (https://eps.leeds.ac.uk/dir-record/research-projects/4265/core-equipment

superstem-2020) and D. McComb (https://profiles.imperial.ac.uk/d.mccomb) are among the world leaders in EELS approach.

There are several important reasons for that:

1) The EELS Atlas provided by Gatan/Ametek company (https://eels.info/atlas) is a reference commonly used in EELS laboratories for an initial rough estimation of elemental edge positions. It is a collaborative resource that does not provide the sources of the data, so it is not possible to determine how the data have been obtained and calibrated. Moreover, EELS atlas does not provide the precise edge positions for a given compound, as these depend on the chemical bonding environment of the element, and not all possible compounds are included in the atlas. For instance, no information is provided for calcium phosphate compounds and data given for calcium are from calcium carbonate with no mention of the phase (calcite or aragonite). A limited number of EELS studies can be found in the literature concerning biominerals and biological systems. In contrast, a large amount of XANES data has been published for these materials, providing reliable information for the different compounds and phases. Edge positions in XANES are exactly the same as EELS ones. This is the reason why this method is usually chosen for this type of study.

2) If ZLP is used for calibration, the ZLP must be recorded in the same spectrum than the edges of interest (carbon and calcium edges at 285 eV and 350eV, respectively). Then, the dispersion has to be chosen to cover the energy range from ZLP to the elemental edges. However, the spectral resolution of an EELS spectrum is not determined by the electron source resolution but also by the dispersion chosen for the acquisition. Our EELS timepix direct detector has 1000 pixels. This requires a dispersion of ≈400 eV which limits the energy resolution to 0.4 eV/px which is not compatible with high spectral resolution analysis of the spectral signatures. In the present study, the dispersion was set to 100 eV to record signal around carbon and calcium edges (285 eV and 350 eV) in order to achieve the best spectral resolution possible.
   An additional reason to use specific peaks for a precise calibration in the energy range of the edges of interest is related to the non-linearity of the energy dispersion of EELS spectrometers.

Firstly, I would like to point out that this is a double-blinded revision so referring to your previous work or groups involved does not mean much - and I'd keep my

comment as unbiased as possible regardless. My doubt partially remains: which peak was used to calibrate? One must have selected one specific peak (the carbonate?) and adjusted its position to calibrate all the remaining peaks. At lines 417-419 I still see a list of peaks mentioned specifically in reference to energy calibration. I am not asking about which peaks were considered for the study in general, but specifically how the calibration was done. I also do not understand why the authors could not use the ZLP with the same acquisition settings and then simply shift the energy range – I understand this cannot be done at once, but, to my knowledge, it could still be done in two steps. And in fact you do shift the energy range eg for Ca and C vs P, as you explain a few answers below. Please also be careful with correct terminology overall both in the manuscript and in these replies: here you're referring to energy dispersion of 100 eV, while I imagine you are actually referring to the energy range, as dispersion is measured in eV/channel and you report 0.1 eV/channel in the manuscript.

It's also not clear which peaks were used since a list of them is mentioned at lines 400-402, while in SI only the carbonate peak is mentioned (lines 11-12 SI).

Answer:

This suggestion has been taken into account. The peaks used for the study are mentioned on the main text at lines 113-120. We had a sentence to specify the position of carbonate peak in the main text at line 118. For greater clarity, we also added an additional mention to the organic peak at 287.5 eV at line 119 and in Figure 2 caption (line 190-191).

2. Core-loss EELS vs. ELNES: this could be an interesting part of the paper but it's placed in SI and the 'carbonate peak' appears in the main manuscript without clear explanations. Overall, I find the presentation and treatment of absorption edges in the core-loss spectrum vs. ELNES peaks (after the C edge, for example the carbonate signature or the 287.5 eV peak used for map-plotting) treated in a confusing manner. Or at least I am presuming that the authors were attempting to do some ELNES, as this is not even clearly mentioned.

Answer:

Part of the explanations given in SI have been transferred to the main text at lines 114-122: "The carbon spectra exhibited a series of sharp peaks associated with

the C 1s → π* transitions, along with a broad shoulder around 300 eV, characteristic for C 1s → σ* transitions. The positions of the sharp peaks vary depending on the carbon bonding environment. This near-edge fine structure can be used to analyze the different compounds. The carbonate is detected on the carbon edge by the presence of a peak at 290.3 eV. As in previous EELS studies concerning biominerals, the distribution of organic compounds was extracted from the peak at 287.5 eV on the C-K edge (cf. SI Appendix, Fig. S1). The phosphorus and calcium maps were obtained from the integration of the corresponding elemental edges after background subtraction, while the different carbon compounds were mapped based on the intensities of their characteristic peaks.".

3. Chosen edges: given the centrality of elemental analyses in the different organelles, I wish the authors also examined other absorption edges especially for P (ie, the K-edge) since the L edge falls within the plasmon tail, which makes it hard to tell if it's not there or just hidden in the background (as the authors also seem acknowledging at lines 209-210). And why not look also at N in addition to C for a better representation of the organics?

Answer:

To the best of our knowledge, the K-edge of phosphorus has not been used previously in the literature for EELS analysis due to its very high energy position (>2100 eV), its signal will be much lower than the P-L_{23} edge and nearly impossible to detect because EELS spectrometers are not designed for these high energies. For instance, the K-edge spectrum is not given in the EELS Atlas (https://eels.info/atlas).

Concerning N, we demonstrated in a previous study that the maps were identical to the ones obtained from the 287.5 eV carbon peak but the signal from N was much noisier than the carbon one due to the low intensity of N signal (Gay et al., 10.1021/acsnano.9b07664).

Another reason is that we tried to minimize the electron dose applied to each area to limit the damage as much as possible. Here, we recorded two spectra on each area: a first one covering the carbon + calcium range (energy range of 100 eV) and a second one for the phosphorus range (energy range of 100 eV). As nitrogen is more than 100 eV distant from carbon, we should repeat a third measurement at the same specimen position, inducing more damage. Alternatively, the energy

range should be increased to 200 eV resulting in a reduction of the energy resolution. Therefore, the optimal approach was to analyze the organic compounds using the carbon edge.

The considerations on P and N reported here could perhaps be mentioned also in the manuscript, eg when you describe the chosen peaks.

4. Overall, I disagree with the terminology ‘Org’ for the EELS maps of the C-edge. They should simply be labelled as ‘C’, similarly to Ca and P, if that element edge is used. I’m not really sure what the chosen C peak really is (287.5 eV) – this should be better discussed, especially in relation to main C edge (see comment 2 for EELS vs ELNES). I’m aware that the authors discuss this in SI, but this is such a key part that it should be also reported in the main manuscript and described in a more convincing manner.

Answer:

There seems to be a misunderstanding here. “Org” is not used to label the entire C-edge signal, but only the small peak at 287.5 eV, which was previously assigned to organic compounds. Please also refer to the answer to question 2 and the corresponding modifications made in the text.

In general, data and images could be better presented. For example:

1. Figure 1: given the importance of different organelles and their locations especially for the model proposed, it would be nice to have consistency in low magnification overview images vs. high magnification insets. It is not clear to me how all these organelles relate to each other, except for 1-I.

Answer:

The low-magnification images in Figure 1 (A-C, J), show the different organelles correctly labelled and clearly localized next to the shell. Low magnification overview images vs. high magnification insets are provided for Figs. 1A-B; 1E-F; 1H-I; 1J-K.

The connection between the mitochondrion and the RAV (Fig. 1-I) is the only direct interaction between vesicles shown in this study and the only one considered in the proposed model.

Some panels contain marked regions that are not shown in the rest of the figure, or at least not labelled like the rectangles in A and J. It is also a bit confusing to include a HAADF-STEM image where the rest of the panel is all BF-TEM.

Answer:

We have taken it into account and we have modified the labels in Figures 1A and 1J to maintain consistency.

The inclusion of an HAADF-STEM image is intended to clearly show the bilayer membranes and the vesicular content of the multivesicular body. Otherwise, in TEM images, the contrast may not be sufficient to clearly reveal these structures.

2. Since the authors want to compare peak intensities, spectra from individual points should be plotted on the same y-axis and not staggered (Figures 2-4).

Answer:

This representation is commonly used in all EELS studies (Nitiputri et al., 10.1021/acsnano.6b02443; Klosowski et al., 10.1038/s41598-018-20072-2; Klosowski et al., 10.1021/acsbiomaterials.6b00300; Ajili et al., 10.1038/s41467-022-29169-9; de Frutos et al., 10.1021/acsnano.2c11169) because it provides a better view of the different spectra.

3. Figure S1-C is missing scale bars!

Answer:

A scale bar (valid for all panels) was added.

4. Why are EELS maps for P not shown in Figures 2 and 3?

Answer:

Maps are not provided for bilayered vesicles and RAV because the signal is much weaker than that of mitochondrial granules and would be lost in the noise. In Figure 4, to obtain a measurable signal, it was summed over a (6×6 px^2) area.

Other aspects that leave me a bit puzzled are:

1. The authors seem quite excited about using a monochromator for getting high

energy resolution data, but I'd like to point out that 150 meV is pretty standard for core-loss EELS – not that I think high spectral resolution matters so much here (and a NION should do better).

Answer:

We strongly disagree with this statement. A resolution of 150 meV in core loss EELS is definitely not accessible without a monochromator. The best electron sources are field emission guns, which provide electrons with an energy dispersion of approximately 300 meV. This value is further degraded to about 600 meV by the point-spread-function of the EELS detector when a CCD is used, as is common in most microscopes. This is for instance the case of the Titan 80−300 microscope at the Imperial College (Kłosowski et al.,10.1021/acsbiomaterials.6b00300). Our monochromated Nion Hermes with the use of a Timepix direct detector provides an energy resolution down to ≈10 meV but only in the EELS low loss range (Chaupard et al, 10.1021/acsnano.2c09571). For this, the dispersion has to be adjusted below 10 eV (0.01eV/px) and this is not compatible with core loss measurements. As a reminder, in this study we used an energy range of 100 eV for the measurements in order to analyse carbon and calcium edges simultaneously.

I am not saying that the monochromator is not useful/needed, nor that a higher energy resolution should be used – I totally agree that this is not feasible here. Hence my point: 150 meV is quite standard for core-loss EELS in monochromated instruments, so it should not really be a 'selling point' for this study, as it seems to be in some part of the paper. But to re-iterate, I don't think very high energy resolution is what matters here, so this is really not an essential discussion point. I think now it works better also considering the re-writing in the comment below.

There also seems to be a bit of confusion regarding spatial vs. energy resolution based on how lines 138-140 are worded.

Answer:

Thanks to the reviewer for the correction of this mistake. The sentence has been rewritten (line 145-148): "the use of monochromated STEM-EELS provides improved spectral resolution and a higher signal-to-noise ratio thanks to the Timepix3 direct electron detector. Combined with the high spatial resolution of

this approach, it enables mapping of different compound distributions at nanometric resolution."

2. Lines 116-117: "Several spectrum images were acquired from each type of organelles". This is not reflected by the content of neither the main manuscript or SI. It's a pity to collect lots of data and then just show spectra from a few points.

Answer:

Additional spectral images for each organelle have been included in the SI (Fig. S2).

3. Line 197: "Identical EELS results were obtained from stained and unstained sections". This is not so well described in the manuscript, and overall it's also unclear whether the samples used for TEM, EDX, or EELS were the same given also the different thickness mentioned in the methodology section.

Answer:

We are confused about this comment. It is clearly stated in the Methods section that we used different section thicknesses for EDX and TEM (100 nm) and for EELS (50 nm). The reason for this is that EELS is not compatible with 100 nm sections, as the signal drops to nearly zero at that thickness due to multiple scattering.

Regarding staining, some sections were stained while others were not. In the unstained sections, only the electron-dense granules were visible and there was no difference in spectra compared to stained sections. Bilayered vesicles and RAV were not visible on unstained sections, so we were obliged to analyse them on stained sections. The good agreement obtained on mitochondrial granules with and without staining demonstrates no influence of staining on EELS data. A sentence has been added at line 208: "EELS analyses of electron-dense granules were performed on stained and unstained sections. No differences were observed in the EELS spectra in both conditions.".

I realize I may sound picky, but the text overall could be a bit smoother not to leave too much to the readers to interpret or figure out. For example, when you describe the preparation of TEM samples for imaging, you clearly mention using an ultramicrotome, while one can only assume you used the same method for preparing the EELS samples? Also the EELS samples are technically also TEM samples, so I guess a better distinction would be samples for imaging vs samples

for EELS. Since you also run a comparison between stained vs unstained, this should be included somewhere, at least in SI. And so what samples are shown in the images and spectra, stained or not? I stand by my comment that these aspects are not really clear.

4. EDX appears a bit randomly and just for one type of organelle.

Answer:

We used EDX mainly to obtain the Ca/P ratio in the electron-dense granules (lines 398-400) because for the other structures the signal was too low to be detected. Because of their high Ca and P content, the EDX maps displayed good resolution, so we found it useful to include them.

5. Overall, the mechanism proposed (represented in Figure 5) seems a bit ambitious based on the experimental evidence.

Answer:

The model, as indicated in line 337 of the main text, is grounded in the results of this study and is consistent with the numerous bone studies cited throughout the manuscript. We consider it highly useful, as it provides a clear schematic representation of our proposal. A clarification has also been included in the caption of Figure 5 (lines 353-354).
